# Long COVID: Deep single-cell immunophenotyping and machine learning reveal a general signature for fatigue

**DOI:** 10.1186/s12967-026-08149-3

**Published:** 2026-04-22

**Authors:** Silke Lauren Sommen, Sunniva Segtnan, Joel Selvakumar, Lise Beier Havdal, Tonje Stiansen-Sonerud, Johannes Gjerstad, Siri Mjaaland, Unni Cecilie Nygaard, Vegard Bruun Bratholm Wyller, Ratnadeep Mukherjee, Lise Lund Berven

**Affiliations:** 1 Department of Pediatrics, Akershus University Hospital, Lørenskog, Norway; 2 University of Oslo, Oslo, Norway; 3 Institute for Clinical Medicine, University of Oslo, Oslo, Norway; 4 Department of Clinical Molecular Biology (EpiGen), University of Oslo and Akershus University Hospital, Lørenskog, Norway; 5 Department of Research and Development in Mental Health, Akershus University Hospital, Lørenskog, Norway; 6 Division of Infection Control, Norwegian Institute of Public Health, Lovisneberggata 8, Room 4361, Oslo, 0456 Norway

**Keywords:** CyTOF, Adolescent, SARS-CoV-2, Post COVID-19 condition, Fatigue, Immunology, Post-infective fatigue syndrome, Long COVID

## Abstract

**Background:**

The post COVID-19 condition, commonly referred to as “Long COVID” (LC), is a constellation of long-lasting and debilitating symptoms following acute SARS-CoV-2 infection, which closely resembles other post-infective fatigue states. The underlying immunological disturbances of LC are poorly understood. Multiple explanatory mechanisms, such as persisting SARS-CoV-2 reservoirs, reactivation of latent viruses, endothelial dysfunction, auto-antibodies, tissue damage, and immune abnormalities, have been proposed but remain incompletely characterized, particularly in younger populations.

**Methods:**

The present study included 12 to 25-year-old females with and without mild SARS-CoV-2 infection, who were prospectively followed for six months after infection and assessed according to the WHO definition of post COVID-19 condition, resulting in four groups (“Long COVID” (LC), recovered convalescents (RC), fatigued controls (FC), healthy controls (HC)). Peripheral blood mononuclear cells were either stimulated with Phorbol-12-myristate-13-acetate/ionomycin or left unstimulated, and analyzed using a 41-antibody CyTOF panel, unsupervised clustering with FlowSOM, dimensionality reduction via UMAP, polyfunctionality assessment with COMPASS, and machine learning for identifying correlates of LC and fatigue. All participants were female with a median age of 18.5 years.

**Results:**

Higher frequencies of Terminal NK cells were associated with LC and FC, showing enhanced polyfunctional responses upon stimulation. Increased CD4 + T cell activation and exhaustion were observed in both LC and FC, with elevated effector memory and effector T cells expressing PD-1, alongside decreased marginal zone B cells and transitional B cells. Machine learning analysis revealed terminal NK cells as the most important feature for predicting fatigue. Importantly, no unique LC-specific immune changes were detected.

**Conclusion:**

Our results point to a shared underlying pathophysiology in LC and other forms of fatigue. The absence of unique LC-related immune changes supports the notion that LC represents a specific example of post-infective fatigue syndrome triggered by SARS-CoV-2 in susceptible individuals. Innate and adaptive immune dysregulation, particularly higher levels of hyperresponsive terminal NK cells, is most striking and could serve as a general fatigue marker rather than an LC-specific biomarker. These findings highlight the need for further studies to correlate immune perturbations with symptom clusters and inform diagnostic and treatment strategies for fatigue states.

**Supplementary Information:**

The online version contains supplementary material available at 10.1186/s12967-026-08149-3.

## Background

Since the beginning of the COVID-19 pandemic, there have been reports of infected adults and children failing to return to baseline health after acute SARS-CoV-2 infection. Ongoing, relapsing, or new symptoms that present 12 or more weeks after the acute phase of SARS-CoV-2 infection and that result in functional impairments, are referred to as “Long COVID” (LC) or “Post COVID-19 condition” (PCC) [1]. Although COVID-19 vaccination and milder presentations of acute SARS-CoV-2 infection have led to a reduced risk of LC [2], as many as 1.7% of the adult US population report significant limitations of their daily activities due to the illness [3]. A recent meta-analysis of persistent symptoms in children and adolescents identified a pooled risk difference of 4% for fatigue at three months post infection in SARS-COV-2 positive compared to controls [4], though estimates can be highly divergent due to heterogeneity in applied clinical case definitions and lack of adjustment for background prevalence of LC symptoms in uninfected individuals. Core symptoms of LC are fatigue, post-exertional malaise, dyspnea, and cognitive dysfunction (“brain fog”), although as many as 200 different symptoms affecting different organ systems have been described and different phenotypes are likely [5]. There is an extensive overlap with symptoms in other post-infective fatigue syndromes (PIFS) [6].

From the time that LC was first noted, efforts to build a pathophysiological framework have been in progress, to some extent building on prior experience of other PIFS. LC might represent a specific example of PIFS triggered by SARS-CoV-2 virus in susceptible individuals and might exhibit similar pathophysiology as in other PIFS patients [7]. Immunological abnormalities are linked to both severe, acute COVID-19 and LC manifestations [8, 9]. Multiple explanatory mechanisms, which are not mutually exclusive, have been proposed [10] and include persisting SARS-CoV-2 reservoirs [11], reactivation of latent viruses [12], endothelial dysfunction [13], auto-antibodies [14], tissue damage [15] and immune abnormalities [15, 16]. Immune dysregulation with low grade inflammation is also commonly reported across PIFS studies (reviewed in: [6]), as are alterations in autonomic nervous activity with sympathetic predominance [17].

Although there is support for immune perturbations in both LC and PIFS, mechanistic coherence is hindered by heterogeneous studies with varying case definitions, lack of control groups and diverse immunological assays. A clear correlation of pathological findings to symptom clusters is also lacking. Additionally, research devoted to elucidation of LC in children and young people is rare, despite high incidence and disease burden [18].

To inform the development of appropriate diagnostic and treatment strategies, well-designed studies and a deep assessment of the immune system are needed. We designed a prospective and strictly defined post SARS-CoV-2 cohort to specifically characterize LC [19]. The aim of the present study was to identify functional immune cell subsets associated with LC by comparing them across four groups: adolescents with LC six months after SARS-CoV-2 infection (LC), adolescents without LC six months after SARS-CoV-2 infection (recovered convalescents, RC), fatigued controls (FC) and healthy controls (HC). Using cytometry by time-of-flight (CyTOF), we achieved broad single-cell immune profiling which allowed us to characterize immune cell subsets associated with LC and fatigue.

## Methods

### Study participants

Participants were recruited through the Long-Term Effects of COVID-19 in Adolescents and Young Adults (LoTECA) study, a prospective, observational cohort study of non-hospitalized adolescents and young adults testing positive or negative for SARS-CoV-2 (ClinicalTrials.gov identifier: NCT04686734). A detailed recruitment procedure and selected data have been reported elsewhere [19–22]. In brief, participants were recruited between December 2020 and May 2021 after undergoing a SARS-CoV-2 Polymerase Chain Reaction (PCR) test. Participants with confirmed positive SARS-CoV-2 polymerase chain reaction (PCR) were designated as SARS-CoV-2 positive (*n* = 405) and patients without confirmed SARS-CoV-2 PCR and negative SARS-CoV-2 serology were designated SARS-CoV-2 negative (*n* = 110). At the time of recruitment, the B.1.1.7 (alpha) SARS-CoV-2 variant was dominant. Clinical data was collected at inclusion and at 6-month follow-up through a one-day investigational program at our research center (Akershus University Hospital, Norway) including a standardized medical assessment, cognitive and respiratory function testing, biological sampling, symptom surveys and a review of medical charts. After the 6-month follow-up, all participants were classified as LC cases/non-cases and PIFS cases/non-cases according to their adherence to two standardized, operationalized definitions of LC: the World Health Organization’s definition of Post COVID-19 Condition (LC [1]), and the Fukuda criteria for post-infectious fatigue syndrome [23]. For this purpose, classification was carried out rigorously, blinded for initial SARS-CoV-2 status and based on evaluation of all collected data and assessment of medical or psychiatric comorbidity [19]. At 6-month follow-up COVID-19 negative participants with self-reported COVID-19 or positive plasma nucleocapsid SARS-CoV-2 antibodies were excluded from further analyses (*n* = 16). COVID-19 vaccination was not routinely offered to adolescents and young adults at the time of recruitment. For all vaccinated subjects, the vaccination took place after the primary COVID-19 infection or after the recruitment, and before sampling at the 6-month follow-up.

For this mass cytometry study, participants (*n* = 80) were selected from four groups (*n* = 20 per group) according to whether they had a prior SARS-CoV-2 infection and whether they were asymptomatic or had post-infective fatigue symptoms. Selection was based on reported fatigue severity (for the affected groups) or absence of symptoms (for the unaffected groups), to achieve better contrasting between the groups. Reported fatigue, a hallmark symptom of LC/PIFS, was recorded using the Chalder Fatigue Questionnaire (CFQ [24], and selection was based on CFQ numerical sores with higher scores pointing to higher symptom burden. By definition, the Long COVID (LC) group was composed of twenty SARS-CoV-2 positive participants with high fatigue score (CFQ $\:\ge\:\:14)\:$and adherence to both the WHO LC and the Fukuda PIFS case criteria. The recovered convalescent (RC) group included 20 SARS-CoV-2 positive participants with uncomplicated recovery, defined by low fatigue scores (CFQ $\:<\:14)$ and non-adherence to LC/PIFS case. Additionally, 40 SARS-CoV-2 negative participants were included as uninfected controls. The uninfected controls were divided into 2 groups: healthy control (HC) group including 20 participants that did not report fatigue and did not adhere to LC/PIFS case definitions; and fatigued control group (FC) including 20 participants that were identified as fatigue-cases (CFQ $\:\ge\:\:14)$. For some individuals in the FC group, a clinical cause could be found for their fatigue and persistent symptoms (Table S1). In most cases fatigue was attributed to adverse life events or psychiatric comorbidity. All selected participants were female. Thus, the experiment included four groups: SARS-COV-2 positive cases with fatigue (LC), SARS-COV-2 positive cases without fatigue (RC), and SARS-COV-2 negative cases with fatigue (FC) and without fatigue (HC). This exploratory study was designed to investigate immunological aberrations in post COVID-19 condition without the intention to develop new diagnostic or therapeutic tools, thus sample size estimation was not included in the study design.

### Blood sampling and PBMC isolation

Two 8 ml BD VacutainerⓇ CPT^™^ (BD, Franklin Lakes, NJ) tubes containing sodium heparin as the anti-coagulant were collected from each participant at the 6-month follow-up appointment. Peripheral blood mononuclear cells (PBMCs) were isolated by centrifugation at 1700 x G for 30 min at room temperature. PBMCs at the interface were collected, rinsed twice with phosphate buffered saline (PBS) and cryopreserved in fetal bovine serum (FBS) with 10% DMSO. All samples were processed within 4 h of collection. Samples were transferred to cryovials and immediately stored at − 80 °C in a CoolCell LX controlled-rate, 2 °C/minute freezing rate, container. After 4–24 h the samples were transferred to -150 °C for long term storage.

### Stimulation assays

PBMC were thawed and seeded in 96–wells plates at a density of 2.2 × 10^6^ cells per well in media supplemented with 10% FBS. Cells were incubated at 37 °C with 5% CO_2_ in a humidified incubator for 20 h prior to 4-hour stimulation. The samples were treated in 2 different conditions: Phorbol myristate acetate/ionomycin (PMA/iono, eBioscience^™^ Cell Stimulation cocktail (500X), 1X, Invitrogen), and medium alone (RPMI, ThermoFisher Scientific) (unstimulated). All stimulations were performed in the presence of protein transport inhibitor (BD GolgiPlug^™^, BD Biosciences) to prevent intracellular cytokine exocytosis. A reference sample was collected from all conditions and samples from the first batch and included in the processing of subsequent batches to allow for batch correction.

### CyTOF antibody staining

The antibodies and channels (Table S2) were selected based on their capacity to identify major immune cell lineages and their subsets, and on the availability of suitable antibodies for CyTOF, either pre-conjugated or after in-house conjugation (with heavy metal isotopes, MaxparⓇ MCP9 and X8 Antibody Labeling Kits, Standard BioTools) and validation during a pilot study (data not shown). The samples were incubated for 10 min on ice with Cell-ID™ Cisplatin viability cocktail (194Pt, Standard BioTools). Next, the samples were incubated with FC receptor blocking solution (Human TruStain FcX^™^, Biolegend) and stained with anti-CD45 antibodies for live cell barcoding employing an approach with combinations of 3 of 6 unique isotopes. The surface antibody cocktail was then added and left to incubate for 30 min at room temperature. Cells were fixed (Maxpar^®^ Fix I Buffer, Standard BioTools), washed and resuspended in ice-cold Methanol for overnight incubation and permeabilization at -20 °C prior to intracellular staining. The following day the cells were rehydrated by washing in Maxpar^®^ Perm-S buffer (Standard BioTools) and incubated with the intracellular antibody cocktail at room temperature (RT) for 30 min. Then, the samples were incubated at RT in 1.6% PFA for 10 min and in Cell ID™ intercalator-IR solution in Fix and Perm Buffer (Standard BioTools) for 20 min before being washed. Finally, the samples were resuspended in freezing medium (FBS with 10% DMSO), combined and transferred to -80 °C until acquisition. The barcoding scheme allowed combination of all stimulation conditions from two participants and a reference sample in one vial.

### Acquisition

Prior to data acquisition on Helios mass cytometer (Standard BioTools), the samples were thawed and washed twice in Maxpar^®^ Water (Standard BioTools). The samples were then diluted in Maxpar^®^ Water (Standard BioTools) containing 10% bead standards (Eq. 4 four element calibration beads, Standard BioTools). Approximately 3 × 10^6^ cell events were collected for each multiplexed sample at an event rate of 250–350 events/second. The mass cytometry data on samples from the 80 participants were acquired in different batches, each acquisition day containing at least one participant from each of the four groups. For batch control, we acquired all samples from one participant simultaneously, we used bead-based normalization and included a reference sample in each batch. The antibody panel, stimulation conditions and staining procedures used in this study were validated by a prior pilot (data not shown).

### Data preprocessing

Raw flow cytometry standard (FCS) data files were normalized using the CyTOF bead-based data normalization software (Standard BioTools). Then, flowrate and signal cleaning was performed using PeacoQC package in R. Next, the files were debarcoded using premessa package in R. Automated Gaussian gating, live/dead gating and doublet removal was carried out using the CyTOFClean package and an in-house R-script. Initial data quality was determined using traditional cytometry statistics and visualization, such as marker expression histograms and heatmaps.

### Clustering and annotations of mass cytometry data

For unsupervised clustering of mass cytometry datasets, we employed FlowSOM [25] using expression of all markers without cytokines. The obtained clusters were then projected onto a Uniform Manifold Approximation and Projection (UMAP) space for visualization. Initially, 30 metaclusters were manually merged by visual inspection of marker heatmaps for CD3, CD19, CD56, and CD11b – to group T cells (CD3^+^), B cells (CD19^+^), NK cells (CD3^-^CD56^+^), and myeloid cells (CD56^-^CD11b^+^). We also observed a small cluster of cells that had an inconclusive marker profile (CD3^+^CD19^+^CD11b^+^). These were termed ‘Other cells’ and not analyzed further. In the secondary clustering, the four principal immune cell types were re-clustered. Manual merging of clusters was performed by marker expression heatmaps/histograms and according to the scheme displayed in Figure S2. For manual annotation we used 20 metaclusters for NK cells and 30 metaclusters for B cells. T cells were further subclustered with 40 metaclusters for CD4^+^ T cells, 40 metaclusters for CD8^+^ T cells and 30 metaclusters for $\gamma \delta$ T cells followed by subsequent merging and annotation. FlowSOM clustering and UMAP dimensionality reduction were performed with the wrapper functions within the *CATALYST* package in R [26].

### Differential analysis of clusters

Comparison of differential abundance of obtained clusters between patient groups was performed by the *diffcyt* package in R. We used the wrapper function available within the *CATALYST* package, setting the testing method to edgeR, which is based on a negative binomial regression model to estimate differences in cluster counts between patient groups [27]. Differences in cluster frequencies were visualized by box-and-whisker plots with scatter overlays. P-values obtained from the differential testing were FDR-adjusted and reported to be statistically significant if adjusted p-values were less than 0.05 for a cell type.

### Polyfunctionality comparison by *COMPASS*

To test for differences in polyfunctional cytokine response induced by stimulation with PMA/iono, we employed the Combinatorial Polyfunctionality Analysis Of Single Cells (*COMPASS*) method [28]. Using a Bayesian framework, *COMPASS* picks cells most likely to have a cytokine response that is specific to the stimulation. Posterior probabilities are calculated for each possible cytokine combination, and the resulting polyfunctionality score can be used to infer differences in response between patient groups. To reduce computation time, we used only the cytokines that responded strongly with PMA/iono stimulation by visually inspecting marker histograms of unstimulated and PMA/iono-stimulated samples, i.e. TNF-alpha (Supplementary Figs. 3, 5, and 7). Differences in polyfunctionality scores between patient groups was assessed by Kruskal-Wallis test followed by Dunn's post-hoc test to adjust for multiple testing. An adjusted p-value < 0.05 was considered statistically significant. *COMPASS* was implemented in R.

### Machine learning

To obtain a minimal set of predictive biomarkers of fatigue, Logistic regression, Linear Support Vector Classifier (SVC), and Random Forest were implemented. Our premise of constructing three different models was to ensure the validity and generalizability of our predictions. Frequencies of the major circulating cell subsets were selected as parameters for the classifiers. 75% of the individuals were selected for the training set, while the remaining 25% were set for model validation. Within the training test, a further 4:1 split was performed to run hyperparameter tuning with 500 iterations and 5-fold cross-validation. The final model run with tuned hyperparameters was then used to predict class labels for the validation set. The entire model from the initial test-train split to prediction was repeated 40 times, and classification accuracy was reported in a confusion matrix as a mean of 40 runs. For assessing the contribution of cell subsets to the overall classification, either feature importance plots (Random Forest) or feature coefficients (Logistic Regression and Linear SVC) were used to display the mean feature score of 40 runs. All machine learning models, and subsequent plotting were implemented in the scikit-learn (1.4.2) package of Python.

## Results

### Study design

**Fig. 1:**
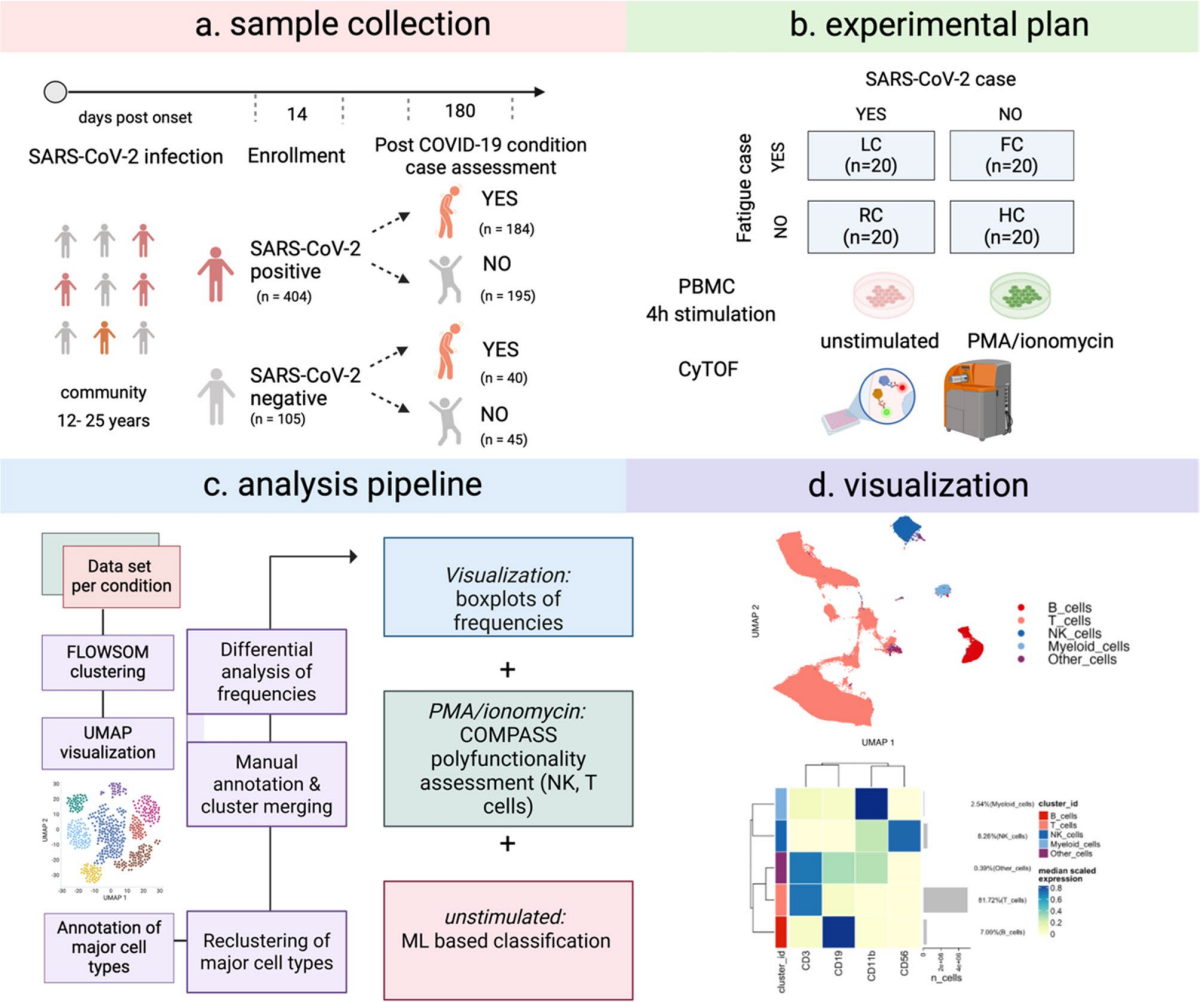
Study design and visualization of CyTOF. (**A**) Study schematic. Peripheral mononuclear cells (PBMCs) were collected from study participants at 6 months after SARS-CoV-2 infection or study inclusion. The participants were divided into four groups dependent on SARS-CoV-2 status and clinical symptomatology at 6 months. (**B**) PBMCs of 20 participants from each group were exposed in vitro to two different conditions: normal cell media (unstimulated) or phorbol myristate acetate (PMA/IONO). The cells were subsequently stained with a 41-antibody mass cytometry panel to identify immune cells and acquired on a mass cytometry platform. (**C**) Mass cytometry data were clustered, visualized using dimensionality reduction methods (UMAP plot), manually annotated based on lineage marker expression and sub-clustered to allow the identification and merging of additional immune subsets. Immune subset frequencies were analysed for differences between experimental groups. Visualisation of significantly different immune cell frequencies were examined by boxplots. Polyfunctionality was assessed using COMPASS. Data corresponding to immune cell subsets of interest were put into machine learning algorithms to define correlates of Long COVID immune phenotype. (**D**) Cells were initially separated into four major clusters annotated as B cells, T cells, natural killer (NK) cells and myeloid cells (UMAP plot). These annotations were based on median expression of known lineage markers across major cluster groups (heatmap plot)

To investigate the underpinnings of LC, we used samples collected during the LoTECA (Long-term effects of COVID-19 in adolescents) cohort of individuals followed systematically for six months after COVID-19 infection [20]. The LoTECA study enrolled 509 participants from December 2021 to May 2021, which were stratified into two study arms: (1) prior SARS-CoV-2 infection and (2) no prior SARS-CoV-2 infection. All participants were invited for a visit in the subacute infection stage and for a visit at six months after infection. Collected data was used to classify participants according to the World Health Organization case definition of PCC at follow-up [19] (Fig. 1A). Classification was done blinded for initial SARS-CoV-2 status and based on reported symptoms, medical history, clinical findings, and routine laboratory assays. For the present study, we used samples from the six-month follow-up that comprises four groups: (a) individuals with persistent symptoms after acute COVID-19 infection (LC, *n* = 20), (b) previously infected individuals without persistent symptoms (RC, *n* = 20), (c) uninfected controls with unspecific fatigue (FC, *n* = 20), and (d) healthy, uninfected controls (HC, *n* = 20) (Fig. 1B). Among the LC and RC groups, all participants had mild acute COVID-19 and the samples for this study were collected, on average, more than six months after acute COVID-19. Median age was 18.5 years, and all participants were female. Additional demographic features and clinical characteristics are shown for each group in Table 1.

**Table 1:** Participant characteristics by experimental group

	Group			
	LC (*n* = 20)	RC (*n* = 20)	FC (*n* = 20)	HC (*n* = 20)
Time since symptom onset/PCR test (days, median (IQR))	211 (13)	213 (12)	208 (18)	209 (13)
**Demographic characteristics**				
Sex (female (%))	20 (100)	20 (100)	20 (100)	20 (100)
Age (median (IQR))	18 (7)	16 (7)	19 (3)	19 (4)
BMI (median (IQR))	21 (6)	21 (3)	22 (4)	22 (5)
Ethnicity (European (%))	12 (60)	14 (70)	20 (100)	18 (90)
Chronic disease^1^ (any (%))	3 (15)	0 (0)	3 (15)	0 (0)
COVID-19 immunization (yes (%))	11 (55)	11 (55)	18 (90)	19 (95)
**Symptoms and functional impairment scores** ^**a**^				
Fatigue^b^ (score 0–33, mean (SD))	25 (4)	10 (2)	17 (4)	11 (3)
Post exertional malaise^c^ (score 0-100, median (IQR))	68 (26)	0 (5)	20 (24)	0 (6)
Cognitive symptoms^d^ (range 4–20, median (IQR)	16 (2.3)	4 (2)	10 (5.3)	5.5 (2)
Respiratory symptoms^e^ (range 3–15, median (IQR))	7.5 (4)	4 (2)	5 (2.3)	5 (2)
Inflammatory symptoms^f^ (range 5–25, median (IQR))	16 (6)	6 (2)	11 (5)	7 (2)
Symptoms of anxiety^g^ (range 0–21, median (IQR))	11 (6)	3 (3.5)	9 (6.8)	4 (3.0)
Symptoms of depression^h^ (range 0–21, median (IQR))	7 (3.5)	1 (3.2)	3.5 (6.25)	1 (3)
Quality of life^i^ (range 0-100, median (IQR))	49 (13)	91 (11)	70 (16)	87 (13)
**Laboratory parameters**				
Hemoglobin (g/dL, median (IQR))	12 (0.7)	13 (1.0)	13 (1.4)	13 (0.8)
Leucocytes (10^9^ g/L, median (IQR))	6.2 (2.2)	5.9 (2.3)	5.7 (1.1)	5.5 (1.5)
Trombocytes (10^9^ g/L, median (IQR))	275 (95)	261 (76)	273 (79)	272 (45)
CRP (high-sensitive µg/mL, median (IQR))	0.9 (1.1)	0.6 (1.2)	2.1 (5.4)	3.5 (4.9)
Ferritine (µg/L, median (IQR))	37 (28)	36 (38)	44 (26)	45 (37)
TSH (mIE/L, median (IQR))	1.7 (1.1)	2.1 (1.0)	1.4 (0.8)	2.0 (0.9)
T4 (pmol/L, median (IQR))	15 (2.9)	15 (3.0)	15(1.8)	15 (3.8)
Kreatinine (µmol/L, median (IQR))	61 (12)	62 (18)	64 (14)	66 (12)
ALT (U/L, median (IQR))	14 (6.5)	15 (4.0)	15 (4.3)	16 (5.5)

Abbreviations: BMI, body mass index, IQR: interquartile range, SD: standard deviation; ^1^self-reported chronic illness; ^a^with the exception of quality of life, higher values imply more symptoms. For quality of life, higher values imply higher quality of life and less functional impairment. ^b^From the Chalder Fatigue Questionnaire. ^c^From the DePaul Symptom Questionnaire. ^d^The sum score across the 4 items memory problems, concentration problems, decision-making problems, and confusion. ^e^The sum of scores across dyspnea, coughing and runny nose. ^f^The sum score across the 4 items headache, muscle ache, fever and fatigue after physical activity. ^g^From the Hospital Anxiety and Depression Scale anxiety subscale. ^h^ From the Hospital Anxiety and Depression Scale depression subscale. ^i^From the Pediatric Quality of Life Inventory

To identify LC-specific immune alterations, peripheral blood mononuclear cells (PBMCs) collected from the study participants at the six-month follow-up visit were used for explorative immunoprofiling by cytometry by time-of-flight (CyTOF) using a 41-antibody panel (Table S2). The PBMCs were either stimulated with Phorbol-12-myristate-13-acetate/ionomycin (PMA/iono) to explore immune cell function or left unstimulated to investigate steady-state immune cell populations (Fig. 1B). Single-cell data from unstimulated and stimulated datasets were explored using an unsupervised clustering approach (FlowSOM) applied to all samples in all groups based on marker expression similarity. Dimensionality reduction visualization by uniform manifold approximation and projection (UMAP) revealed groupings for clusters characterized by expression of known lineage markers of major immune cell populations: T cells, B cells, NK cells and myeloid cells (Fig. 1C-D). We did not find any differences in frequencies for these major cell subsets between the experimental groups (Figure S1). To resolve additional immune subsets, each of these major cell types was further sub-clustered, annotated, and analyzed for differences in cell population frequencies between the groups. COMPASS, an algorithm that models observed cell subsets displaying different protein marker combinations to present polyfunctional subsets that occur in stimulated cells, in reference to an unstimulated control condition, was used to explore cell-subset responses to stimulation for a more comprehensive understanding of cellular function [28]. Finally, the immune cell frequencies were put into machine learning algorithms to define correlates of the LC and unspecific fatigue immune phenotypes (Fig. 1C).

### Higher frequencies of CD56^lo^CD16^hi^ terminal NK cells are associated with long COVID and nonspecific fatigue

**Fig. 2:**
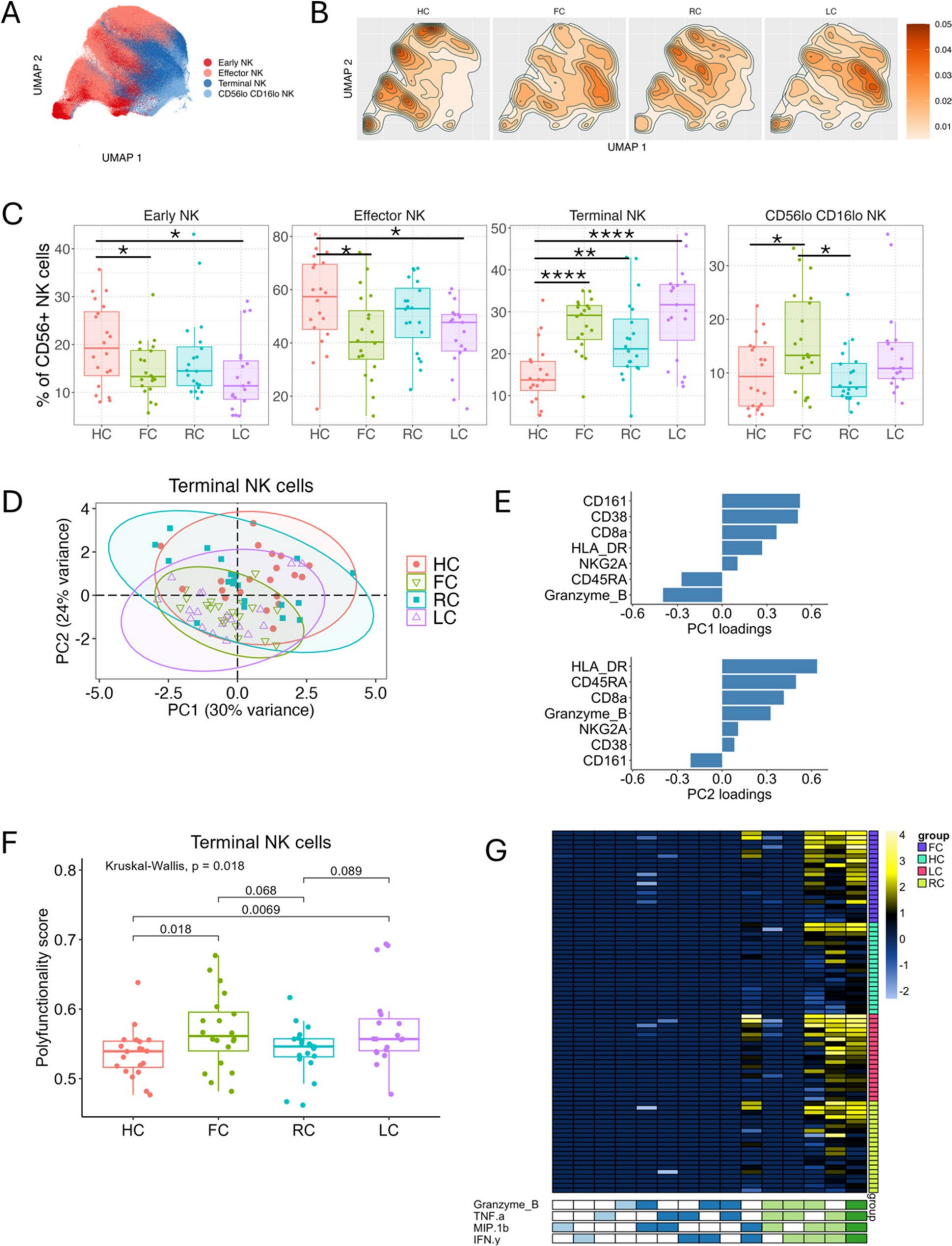
NK cells. (**A**-**B**) UMAP of NK cells at 6 months, colored by NK major cell subset (S), and colored by cell density for each experimental group (HC, FC, RC, LC) (**B**). (**C**) Boxplots with individual levels (dots) display NK cell subset frequencies (% of total CD56 + NK cells) in each group (LC, RC, FC, HC). Negative binomial distribution was used to evaluate differences in mean frequencies in pairwise comparisons between groups. P-values were FDR-adjusted using Benjamini-Hochberg correction. Horizontal bars show statistically significant differences between two groups with *, **, ***, **** = p values of < 0.05, 0.01, 0.001, 0.0001. Box plots: upper, lower and center box lines represent upper quartile, lower quartile and mean. (**D**) PCA plot of terminal NK cell phenotypic heterogeneity, per NK cell subset, colored per group (red = HC (*n* = 20), green = FC (*n* = 20), blue = RC (*n* = 20), purple = LC (*n* = 19) with 95% confidence ellipse. (**E**) Loadings of principal components PC1 and PC2 contributing to overall variability in PCA for the terminal NK cells in D. (**F**) Polyfunctionality calculated by COMPASS for each sample’s response to PMA/iono stimulation. Box plots of polyfunctionality score (PFS) in HC (red), FC (green), RC (blue), LC (pink) in response to PMA/iono-stimulation. Pairwise differences were based on Kruskal-Wallis test followed by Dunn’s post hoc test. Horizontal bars show statistically significant differences between two groups. (**G**) COMPASS heat map displaying terminal NK cell response to PMA/iono in all four groups. In the heat map, columns correspond to the different cell subsets in which responses were detected and are color-coded by the cytokines they express (white = “off”, colored = “on”), grouped by their degree of polyfunctionality, and are displayed in order of increasing polyfunctionality from left to right (sky blue = 1, dark blue = 2, and light green = 3). Rows represent study subjects, which are ordered by their group: FC (purple), HC (turquoise), LC (pink), RC (green), and by PFS within each group. Each cell of the heatmap shows the probability estimated by COMPASS that the observed response is PMA/iono-specific in the corresponding sample (row) and cell subset (column), where the probability is color-coded from white (zero) to yellow (one)

NK cells were identified as CD3^−^ CD19^−^ CD56^+^, sub-clustered (k = 20) and merged into subsets based on CD56 and CD16 surface marker expression: CD56^hi^ CD16^lo^ early NK cells, CD56^hi^ CD16^hi^ effector NK cells, CD56^lo^ CD16^hi^ terminal NK cells, CD56^lo^ CD16^lo^ NK cells (Fig. 2A, Figure S2A, S3A). Intergroup differences in NK cell subsets were apparent when projecting cell density into two dimensions by uniform manifold approximation (UMAP) with an expanded CD56^lo^ CD16^lo^ NK cell subset in LC and FC (Fig. 2B). CD56^lo^ CD16^lo^ NK cells were indeed significantly increased in FC compared to HC and RC, but the comparison did not reach statistical significance for LC group (Fig. 2C). Similarly, LC and FC groups showed higher frequencies of terminal NK cells than the HC group (Fig. 2C). Early NK and effector NK cells had significantly lower frequencies in the FC and LC groups than the HC group (Fig. 2C). In the RC group, terminal NK cells were more abundant compared to the HC group and CD56^lo^ CD16^lo^ NK cells were reduced compared to the FC group, while all other NK cell subsets did not statistically differ from the other groups, suggesting an intermediary position of RC NK cell subset frequencies between HC and LC/FC groups (Figs. 2B-C). Strikingly, none of the subset frequencies differed between the LC and FC groups (Fig. 2C, Table S3). Next, we applied principal components analysis (PCA) to the marker expression of the terminal NK cell subset to explore sources of variation in the experimental groups. Projection of the samples in PCA space revealed discrimination of LC and FC samples compared to HC and RC samples along principal component 2 (PC2) (Fig. 2D). The factor loadings of PC2 showed that the phenotypic separation was driven by positive contributions of activation markers HLA-DR and opposite contributions of inhibitory marker CD161 to PC2 (Fig. 2E). To determine if immune cell activation was affected in these four NK cell types after resolution of the acute SARS-CoV-2 infection, we stimulated the cells using PMA/iono. We observed increased intracellular production of Granzyme B, IFN-γ, MIP-1β, and TNF-α in NK cell subsets from all groups (Figure S3B) and identified differences in response profiles in the NK cell data by PCA (Figures S4A-C). Differences in subject- and NK cell population-specific responses were interrogated by COMPASS, a tool for analyzing polyfunctional cytokine response, i.e. the complexity and quality of immune responses post stimulation. Intergroup differences were observed in terminal NK cells only, where FC and LC had higher polyfunctionality scores than RC and HC, indicating phenotypically altered NK cells with enhanced production of multiple effector cytokines upon PMA/iono-stimulation (Fig. 2F, Figure S4D-F). The increased polyfunctional response in terminal NK cells of LC and FC compared to RC and HC subjects includes combinations of 2, 3 and 4 cytokines (Fig. 2G). Thus, our results reveal the presence of significantly higher relative proportion of terminal NK cells with a concurrent decrease in early and effector subsets at steady-state with a higher potential to produce a polyfunctional response upon stimulation in subjects with fatigue compared to asymptomatic subjects.

### Differences in T cell subset frequencies and cytokine functionality distinguishes long COVID from convalescent individuals

**Fig. 3:**
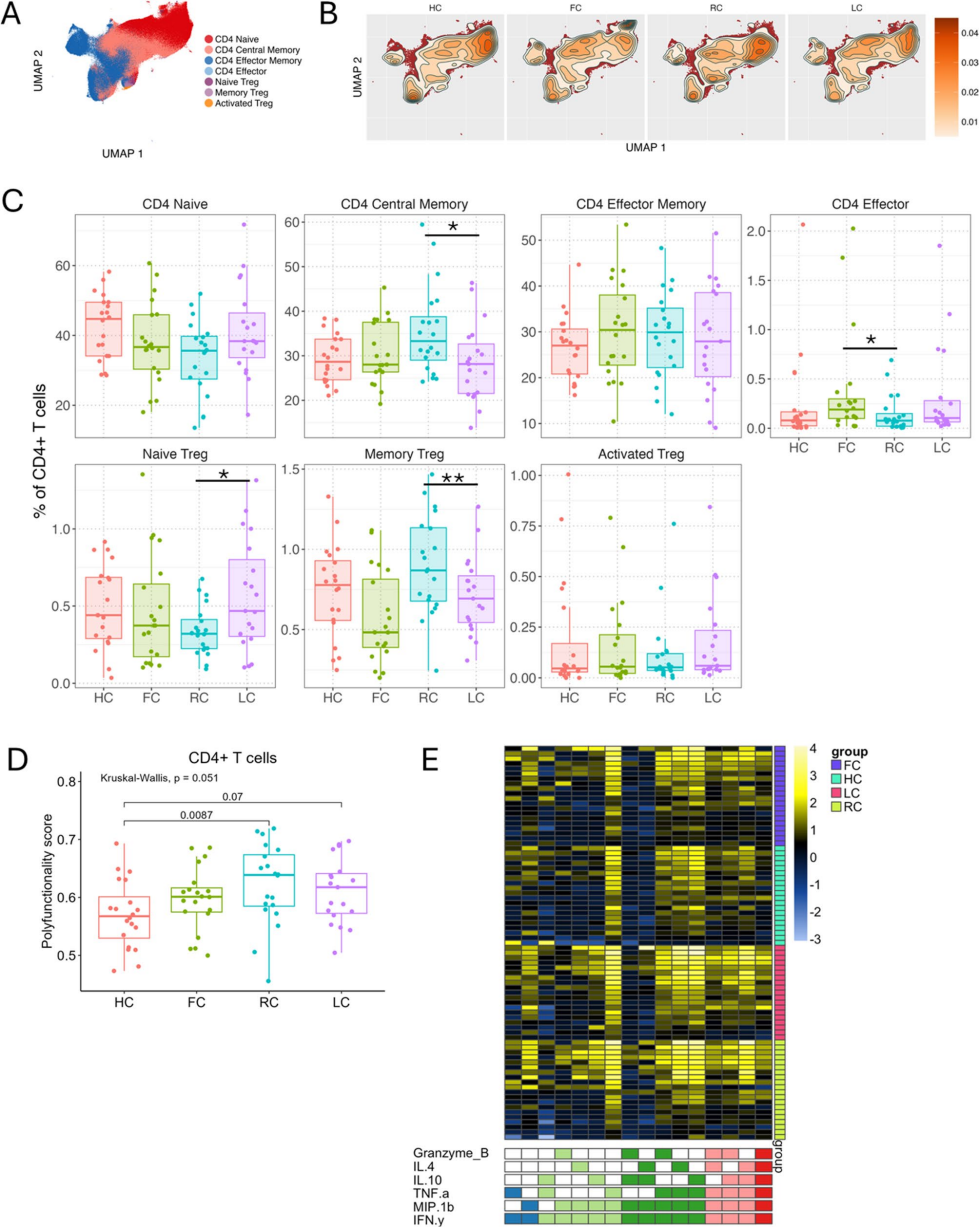
CD4 + T cells. (**A**-**B**) UMAP of CD4 + T cells at 6 months, colored by CD4 + T cell subset (A), and colored by cell density for each experimental group (HC, FC, RC, LC) (**B**). (**C**) Boxplots with individual levels (dots) display CD4 + T cell subset frequencies (% of total CD3+/CD4 + T cells) in each group (LC, RC, FC, HC). Negative binomial distribution was used to evaluate differences in mean frequencies in pairwise comparisons between groups. P-values were FDR-adjusted using Benjamini-Hochberg correction. Horizontal bars show statistically significant differences between two groups with *, **, ***, **** = p values of < 0.05, 0.01, 0.001, 0.0001. Box plots: upper, lower and center box lines represent upper quartile, lower quartile and mean. (**D**) Polyfunctionality calculated by COMPASS for each sample’s response to stimulation. Box plots with individual levels (dots) of polyfunctionality score in HC (red), FC (green), RC (blue), LC (pink) in response to PMA/IONO-stimulation. Pairwise differences were based on Kruskal-Wallis test followed by Dunn’s post hoc test. Horizontal bars show statistically significant differences between two groups. (**E**) COMPASS heat map displaying CD4 T cell response to PMA/iono in all four groups. In the heat map, columns correspond to the different cell subsets in which responses were detected and are color-coded by the cytokines they express (white = “off”, colored = “on”), grouped by their degree of functionality, and are displayed in order of increasing functionality from left to right (dark blue = 2, light green = 3, dark green = 4, and pink = 5). Rows represent study subjects, which are ordered by their group: FC (purple), HC (turquoise), LC (pink), RC (green), and by PFS within each group. Each cell of the heatmap shows the probability estimated by COMPASS that the observed response is PMA/IONO-specific in the corresponding sample (row) and cell subset (column), where the probability is color-coded from white (zero) to yellow (one)

Analysis of circulating CD4^+^ T lymphocyte populations revealed no differences in naïve (CD45RA^+^CCR7^+^), effector memory (Tem, CD45RA^−^CCR7^−^), and activated regulatory T cell (CD25^+^CD127^−^HL-DR^+^CD45RA^−^) subsets after merging of FlowSOM clusters and manual annotation of major CD4^+^ cell subsets (Figs. 3A-B, Figure S2B). On the other hand, central memory (Tcm, CD45RA^−^CCR7^+^), effector (Teff, CD45RA^+^CCR7^−^), Naïve Treg (CD25^+^CD127^−^HL-DR^−^CD45RA^+^), and memory Treg (CD25^+^CD127^−^HL-DR^−^CD45RA^−^) populations showed significant differences between the RC and LC groups (Fig. 3C). Individuals belonging to the LC group showed higher frequencies of Naïve Treg population, while demonstrating a concurrent lower in the frequencies of Memory Tregs and CD4^+^ central memory cells as compared to the RC individuals (Fig. 3C). There was also an observable trend of increased frequencies of CD4^+^ effector T cells in both the fatigue groups compared to HC and RC groups, although only the difference between FC and RC was statistically significant at *p* < 0.05 (Fig. 3C). Assessment of PMA/iono-stimulated functional response within the major CD4^+^ populations for expression of five key cytokines (Granzyme B, IL-10, TNF-α, MIP-1β, IFN-γ) demonstrated significantly enhanced polyfunctional response in CD4^+^ T-cells of recovered convalescents (Fig. 3D-E) as compared to healthy controls (HC), while the two fatigue groups were more similar to the healthy controls (Fig. 3D-E).

**Fig. 4:**
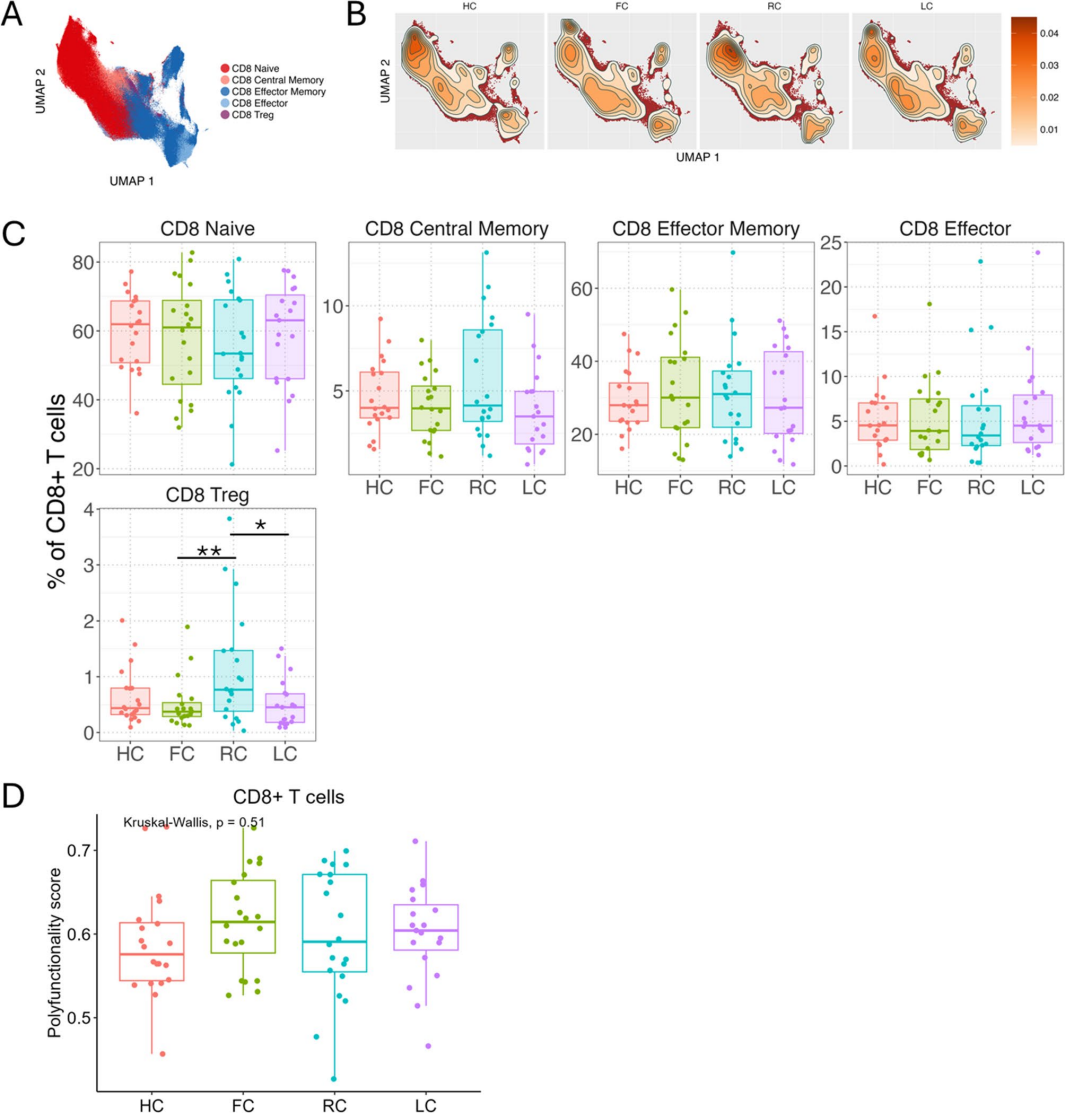
CD8 + T cells. (**A**-**B**) UMAP of CD8 + T cells at 6 months, colored by CD8 + T cell subset (**A**), and colored by cell density for each experimental group (HC, FC, RC, LC) (**B**). (**C**) Boxplots with individual levels (dots) display CD8 + T cell subset frequencies (% of total CD3+/CD8 + T cells) in each group (LC, RC, FC, HC). Negative binomial distribution was used to evaluate differences in mean frequencies in pairwise comparisons between groups. P-values were FDR-adjusted using Benjamini-Hochberg correction. Horizontal bars show statistically significant differences between two groups with *, **, ***, **** = p values of < 0.05, 0.01, 0.001, 0.0001. Box plots: upper, lower and center box lines represent upper quartile, lower quartile and mean. (**D**) Polyfunctionality calculated by COMPASS for each sample’s response to stimulation. Box plots with individual levels (dots) of polyfunctionality score in HC (red), FC (green), RC (blue), LC (pink) in response to PMA/IONO-stimulation, by CD8 + T cells. Pairwise differences were based on Kruskal-Wallis test followed by Dunn’s post hoc test

FlowSOM clustering followed by cluster merging and annotation merged into five distinct subsets within the CD8^+^ T cell compartment – Naïve (CD45RA^+^CCR7^+^), Central Memory (CD45RA^−^CCR7^+^), Effector (CD45RA^+^CCR7^−^), Effector Memory (CD45RA^−^CCR7^−^), and CD25^+^CD127^−^ CD8 Tregs cells (Fig. 4A-B, Figure S2C). The abundances of most of the major CD8^+^ T cell subsets were comparable between the study groups (Fig. 4C). However, the recovered convalescent individuals demonstrated significantly higher abundances of CD8^+^ Tregs compared to both FC and LC groups (Fig. 4C). As in the case of CD4^+^ T cells, activation-induced CD8^+^ T cell response was analyzed using COMPASS for expression of seven key cytokines (Granzyme B, IL-2, IL-4, IL-10, TNF-α, MIP-1β, IFN-γ) (Figure S7). No differences in polyfunctionality score for CD8^+^ T cells were found between any of the groups (Fig. 4D). A similar analysis of gδT cell subsets did not reveal any statistically significant differences between the four groups (Figure S2E, Figure S6).

### Decreased marginal zone B and increased double negative B cells further distinguish fatigue states

**Fig. 5:**
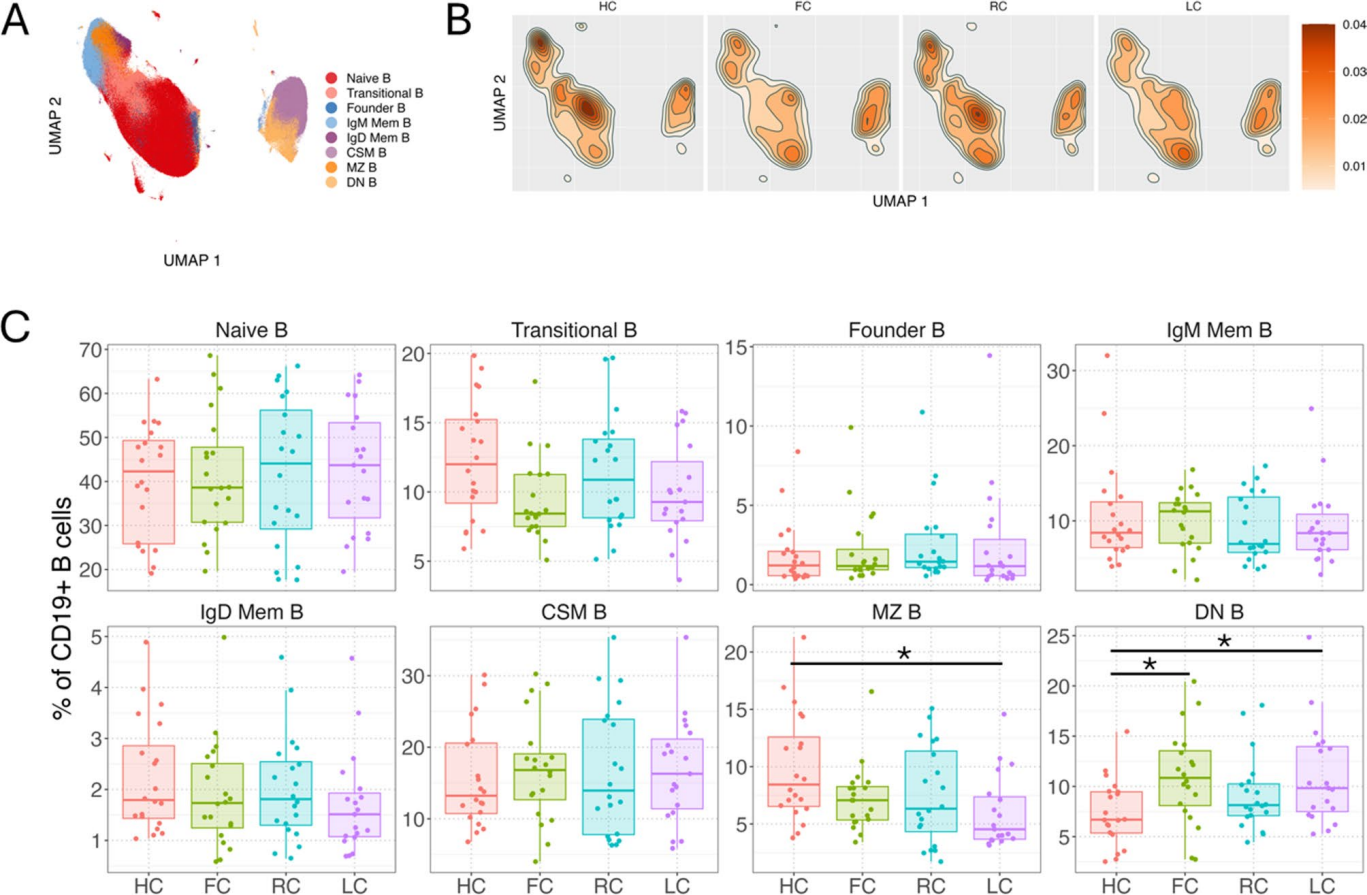
B cells. (**A**-**B**) UMAP of B cells at 6 months, colored by B cell subset (**A**), and colored by cell density for each experimental group (HC, FC, RC, LC) (**B**). (**C**) Boxplots with individual levels (dots) display B cell subset frequencies (% of total CD56 + NK cells) in each group (LC, RC, FC, HC). Negative binomial distribution was used to evaluate differences in mean frequencies in pairwise comparisons between groups. P-values were FDR-adjusted using Benjamini-Hochberg correction. Horizontal bars show statistically significant differences between two groups with *, **, ***, **** = p values of < 0.05, 0.01, 0.001, 0.0001. Box plots: upper, lower and center box lines represent upper quartile, lower quartile and mean

A representative plot depicting the stepwise strategy to merge B cells into eight functional B cell subsets after sub clustering is shown in Figure S2D. Intergroup differences in B cell subsets were apparent when projecting cell density into two dimensions by uniform manifold approximation (UMAP) (Fig. 5A-B). Levels of transitional B (CD27^−^IgD^+^CD24^+^CD38^+^) cells demonstrated a trend of decreased frequencies in FC and LC as compared to HC (Fig. 5C), however the result was not statistically significant. Marginal Zone B (CD27^+^IgM^+^IgD^+^) cells were decreased in both LC and FC compared to HC, while Double Negative B (IgD^−^CD27^−^CD24^−^CD38^−^) cells were increased (Fig. 5C). Circulating levels of naïve B cells and other B cell subsets were not found to be significantly different between the groups (Fig. 5C).

### Machine learning reveals a general immune cellular signature of fatigue

**Fig. 6:**
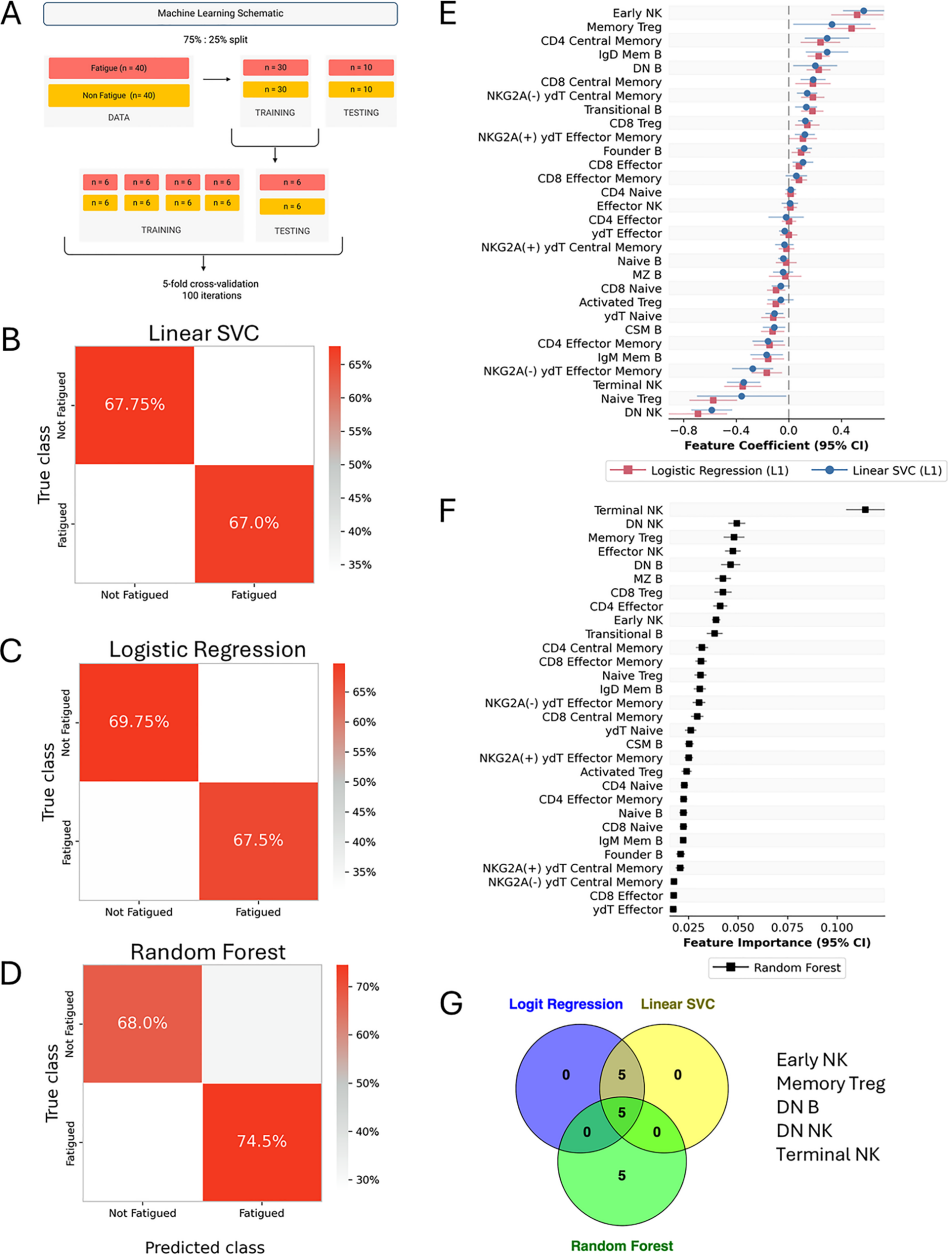
Identifying a common signature of fatigue by machine learning. (**A**) Schematic showing data splitting into training and test sets, and the strategy for cross validation. (**B**) Confusion matrix showing classification accuracy achieved by Linear SVC models. Average accuracy scores for 40 iterations of the pipeline shown in (**A**) are displayed here. (**C**) Confusion matrix showing classification accuracy achieved by Logistic Regression models. Average accuracy scores for 40 iterations of the pipeline shown in (**A**) are displayed here. (**D**) Confusion matrix showing classification accuracy achieved by Random Forest models. Average accuracy scores for 40 iterations of the pipeline shown in (**A**) are displayed here. (**E**) Forest plot depicting feature coefficients obtained from Linear SVC and Logistic Regression models. (**F**) Variable importance plot (VIP) showing immune cell subsets and their importance for prediction accuracy obtained by Random Forest. (**E** and **F**) Error bars represent the 95% C.I. of the average feature importance score from 40 iterations of the models. (**G**) Venn diagram showing the top 5 common important features shared among the three models

Since we did not find any differences in immune cell frequencies between FC and LC (Table S3) and several of the observed differences seemed to be common for the two fatigue groups, we sought to investigate if frequencies of immune cell types are predictive of a general fatigue condition, irrespective of infection status. To this end and to identify a minimal set of biomarkers associated with fatigue, the cell frequencies were used to train classifiers to predict one of healthy (HC), convalescent (RC) and fatigue (FC + LC) categories (Figure S8). Our initial training with a Random Forest classifier demonstrated 80.8% prediction accuracy for the fatigued cases, however a large proportion of the convalescent individuals were misclassified as fatigued, with only 46% healthy individuals classified correctly (Figure S8A). Inspection of feature importance scores revealed Terminal NK cells to be the most important feature distinguishing the classes (Figure S8B). We reasoned that the high rate of misclassification in the convalescent and healthy classes could be due to the presence of a high number of noisy features. Therefore, we trained an L1-penalized Linear Support Vector Classifier to automatically reduce the number of noisy features. This led to a significant improvement of classification accuracy for the convalescent individuals (51.81%, Figure S8C). Overall, the Linear SVC performed better class discrimination compared to the Random Forest, with NK cell subsets contributing most to between-class separation (Figure S8B, D). Importantly, both the models performed poorly in predicting the healthy controls and convalescent individuals. Therefore, we decided to group them together to predict the fatigue state (regardless of infectious trigger) from individuals without fatigue-like symptoms (Fig. 6A). Our results with Linear SVC show good classification accuracy to predict fatigued cases (67%) from non-fatigued ones (67.5%) (Fig. 6B). To validate our prediction accuracies, we then trained L1-penalized Logistic Regression (Fig. 6C) and Random Forest classifiers (Fig. 6D), both of which showed very good agreement with the Linear SVC model. We then examined the feature coefficients (Linear SVC and Logistic Regression) to identify the most important features contributing to the class separation. This revealed Early NK, Memory Tregs, CD4 Central Memory, IgD Mem B, and DN B cells as the top five positive features, while DN NK, Naïve Treg, Terminal NK, NKG2a(-) ydT Effector Memory, and IgM Mem B cells to be the top five negative coefficients (Fig. 6E). Inspection of the feature importance ranks obtained from the Random Forest model revealed the Terminal NK cell frequencies to be by far the most important predictive feature (Fig. 6F). Finally, we wanted to find out the common important features of the three separate models. The Venn diagram in Fig. 6G demonstrates that frequencies of terminal, early and DN NK cell subsets were the most important predictive features of a fatigued state in addition to memory Tregs and DN B cells.

## Discussion

Here, we present the results of a mass cytometry study investigating immune correlates of fatigue complaints after SARS-CoV-2 infection. The main findings from this study are: (a) Absence of unique LC-related immune changes; and (b) Innate and adaptive immune dysregulation associated with persistent fatigue irrespective of triggering event, of which higher levels of terminal NK cells displaying hyperresponsiveness to stimulation (higher cytotoxic cytokine production) is most striking.

NK cells are important in the recognition and elimination of SARS-COV-2, and multiple studies have reported changes in this innate immune population in acute COVID-19 (reviewed in [29]). The role of NK cells in LC is less clear with conflicting evidence ranging from elevated effector (CD56 + CD16+) NK cells with higher expression of activation marker NKG2C [30], to no differences [31] or even decreased NK cells [32, 33]. Studies in other persistent fatigue conditions report attenuated NK cell cytotoxicity [34] alongside NK cell subset changes [35–38]. In contrast, our results argue for enhanced cytotoxic potential since terminal NK cells are known to be primarily responsible for cytotoxic activity and cytokine release [39]. Importantly, we find similar alterations in NK cell populations in LC and fatigue resulting from other causes. After exposure to an infectious agent, early NK cells differentiate to effector and terminal NK cells in response to the infectious trigger. After resolution of the acute infection, a return to homeostasis with normalization of NK cell subset frequencies is expected. However, our findings suggest a disruption of this traditional pathway of differentiation in fatigued individuals, where terminal NK cell frequencies remain elevated and are indicative for an incomplete restoration of NK cell homeostasis. In the case of acute SARS-CoV-2 infection, Ryan et al. [40] have shown that certain signatures of active infection can persist well into COVID-19 convalescence and resolving transcriptional dysregulation is observed in recovering convalescents but not in LC patients. In line with this, recovered convalescents in our study show an intermediary NK cell subset distribution between fatigued individuals and healthy controls. Consistent with a study by Galan et al. [30], we highlight the importance of NK cells in our machine learning classification models of fatigue. Of importance, our model poorly discriminates LC from FC cases, showing that relative abundance of NK cell subsets cannot be used as LC-specific biomarker, but could possibly serve as a general fatigue marker.

Assessment of the T cell subsets revealed higher frequencies of Effector CD4 + T cells together with decreased frequencies of CD4 + Central Memory T cells in the two fatigued groups as compared to convalescent individuals. Memory T cells from individuals affected with Long COVID often show decreased proliferative capacity and increased expression of homing receptors, demonstrating a preferential migration out of blood to inflamed tissue [31]. Elevated T effector memory cells after cytokine directed proliferation has been reported in PIFS after infectious mononucleosis and aligns with signs of T cell activation and chronic inflammation [41]. Our data additionally shows an increase in naïve regulatory T (Treg) cell frequencies in LC, in line with earlier findings in LC and other fatigue states [30, 31, 42, 43]. Treg cells are known to quench inflammation upon resolution of an immune response. So, while altered balances in the central memory and effector T cells may suggest failure to control persistent immune activation, the increase in Tregs could point to an attempt to restore homeostatic balance and limit chronic inflammation. Interestingly, however, we observed higher cytokine polyfunctionality in CD4 + T cells of convalescent individuals compared to healthy controls, while the fatigued groups were comparable. This may indicate better capacity of viral clearance in the RC group, that may reduce the chance of viral persistence following recovery, a phenomenon that has been linked to development of post-infectious fatigue, especially in the context of SARS-CoV-2 infection [44]. In the B cell compartment, we discovered increased double negative B cells in fatigued groups as previously reported in LC and other auto-inflammatory conditions (e.g. SLE) [16, 45], and decreased marginal zone B cells. We also see a trend of lower transitional B cell frequencies, possibly caused by abnormal T cell mediated signals affecting B cell differentiation. Similar changes in B cell survival and differentiation have been previously reported in other fatigue syndromes [46, 47], although findings are divergent with others reporting increases in immature B cells and memory B cells [42].

An unexpected finding was the apparent lack of unique LC-related immunological changes in relation to other fatigue states, indicating that the observed immune profile is associated to SARS-CoV-2 infection, but is not SARS-CoV-2 specific. By showing identical immune cell subset changes in both fatigued groups, we provide strong indications that LC is a subtype of PIFS rather than a single condition. Instead of considering immune dysregulation specific to SARS-CoV-2 virus central to LC pathophysiology [48, 49], in light of the present results and earlier reports of altered autonomic nervous control of innate immunity [50], we propose a framework in which the infectious trigger is not the only driver of fatigue-associated immune changes. Initial viral-triggered immune activation can cause fatigue as part of the acute sickness response [51], and recovery after infection is associated with resolution of symptoms and with slow immunological return to homeostasis, as shown by the intermediary immunophenotype of the RC group in the present study. Persistent fatigue, however, could be the result of a cascade of effects after the triggering event, i.e. SARS-CoV-2 infection in LC, associated with long-lasting immune changes. Interestingly, studies in PIFS have indicated a poor correlation between these immune alterations and persistent symptoms [52–54], but have found associations between autonomic markers and inflammatory markers [54]. Sympathic predominance has been repeatedly reported in PIFS [17, 54] and is known to influence the immune system by promoting proinflammatory gene transcription and cytokine production via beta-adrenergic receptor actions [55, 56]. Potentially, autonomic alterations in persistently fatigued individuals could explain a common immune profile across different infectious triggers as well as other precipitating events. Either way, our data corroborates previous studies suggesting that LC and PIFS are closely related and share a common pathophysiology [6, 57].

Our study has some important limitations. We describe observations in peripheral blood mononuclear cells, and consequently could not assess changes in granulocyte populations or tissue-resident cells, as well as myeloid cells, the frequencies of which can be highly unreliable in frozen PBMCs. We are also limited by a relatively small sample size. However, this is partially mitigated by using samples from a large, prospective, and phenotypically well-characterized cohort. Discrepancies of our findings with earlier studies might reflect the heterogeneity of the condition (depending on applied clinical case definition, viral variant, acute disease severity, time point after infection, vaccination/re-infection and gender), which could affect immune responses, or the absence of appropriate control groups (e.g. COVID-19 convalescents, uninfected individuals) in other studies. The current study only focused on female adolescents and young adults, and the generalizability of our findings to males and older adults is uncertain. Moreover, owing to the small sample size, effects of potential confounders (chronic disease, vaccination) cannot be reliably estimated. However, our goal in this manuscript was not to establish a causal relationship to the observed outcomes but to obtain immunological signatures of fatigue. Further studies with larger cohorts are needed to establish causal associations of clinical, immunological, and demographic data with the observed outcomes. The results presented in this study are based on a single timepoint analysis. Consequently, mechanistic and longitudinal studies are required to explore the basis of the changes we observe and to explain what occurs in the transition stage from acute infection to persistent fatigue development. Another potential limitation of our study is the use of PMA/Ionomycin, which is a rather strong activator. Our aim for using it in this study was to assess global activation potential of a wide range of cell types and compare those between the study categories. Future research endeavours could be geared towards more nuanced assessment of subset-specific functions. Many knowledge gaps remain to be filled, and based on the sensitivity of the present approach, we advocate for the use of high- dimensional single-cell analyses in future studies of mechanisms and biomarkers of these conditions.

## Conclusions

In the present study, we provide empirical evidence of the similarities in the circulating immune cellular ecosystems of patients displaying post COVID-19 symptoms of chronic fatigue with those of patients suffering from chronic fatigue symptoms of unknown origin. Our results indicate towards an underlying immune disturbance that could be specific to the fatigue condition, irrespective of its etiology. This has extremely important implications for future design of studies with larger cohorts targeted at finding biomarkers associated with symptoms of chronic fatigue.

## Supplementary Information

Below is the link to the electronic supplementary material.

Supplementary Material 1

## Data Availability

The data analysed in this study come from a cohort of human volunteers and therefore contains information that may be perceived as sensitive. Due to protection of privacy and restrictions from the Norwegian Data Inspectorate and the Regional Committee for Medical and Health Research Ethics, the data are not publicly available. However, to facilitate reproduction of our analysis, encoded raw fcs files without attached metadata can be made available upon reasonable request to the corresponding author. All computer codes for the analysis of data reported here are part of publicly available packages and are cited in the methods section. The work does not generate new codes for analysis. R and Python scripts used for data analysis can be made available upon request to the corresponding author.

## Abbreviations

LoTECA: Long-Term Effects of COVID-19 in Adolescents and Young Adults
PCC: Post COVID-19 condition
PIFS: Post-infective fatigue syndrome
CFQ: Chalder Fatigue Questionnaire
IFN: Interferon
PMA: Phorbol-12-myristate-13-acetate
CyTOF: Cytometry by Time-of-Flight
COMPASS: Combinatorial Polyfunctionality Analysis Of Single Cells
PCA: Principal component analysis
SOM: Self organizing map
SVC: Support vector classifier
SLE: Systemic lupus erythematosus
