# Supplementary material for: Long COVID: Deep single-cell immunophenotyping and machine learning reveal a general signature for fatigue
Source: J Transl Med. 2026 Apr 22;24:736. doi: 10.1186/s12967-026-08149-3 (PMC13237906; doi:10.1186/s12967-026-08149-3)
Supplement: Supplementary file 1 — Supplementary Material 1 [file 12967_2026_8149_MOESM1_ESM.pdf]

Additional file

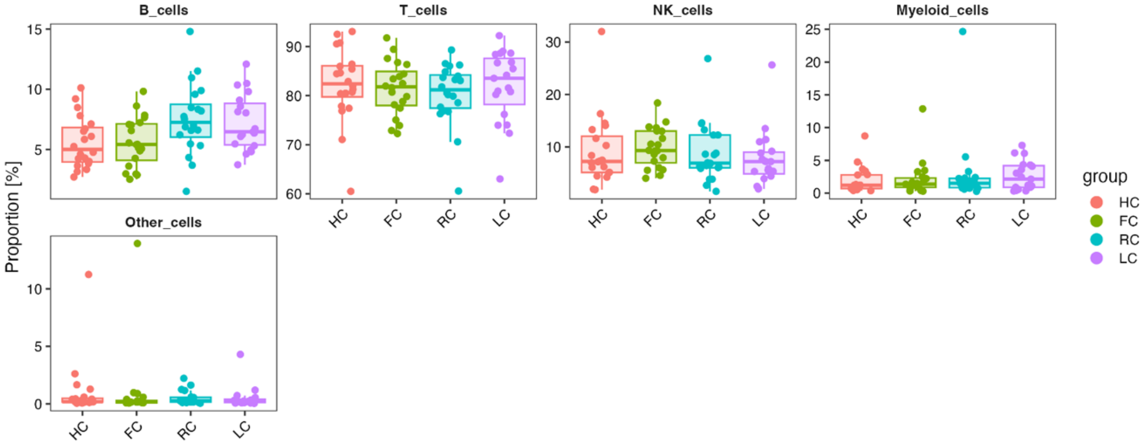

Figure S1. Frequencies of major cell types. Boxplots with individual levels (dots) display major cell subset frequencies (% of total cells) in each group (LC, RC, FC, HC). Negative binomial distribution was used to evaluate differences in mean frequencies in pairwise comparisons between groups. P-values were FDR-adjusted using Benjamini-Hochberg correction. Horizontal bars show statistically significant differences between two groups with *, **, ***, **** = p values of < 0.05, 0.01, 0.001, 0.0001. Box plots: upper, lower and center box lines represent upper quartile, lower quartile and mean.

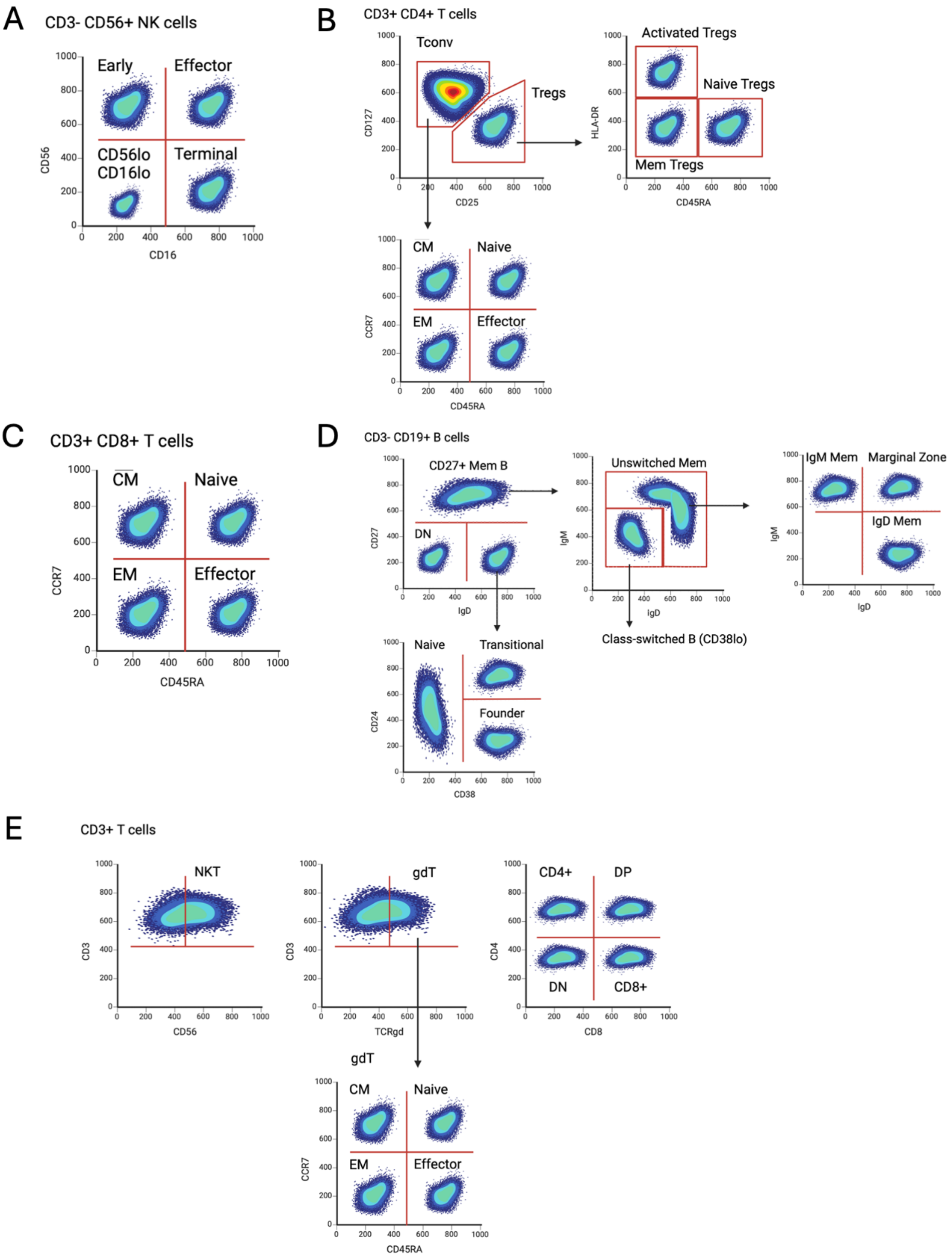

Figure S2. Annotation strategy of major cell types and subsets. Live cells were split into subsets based on expression of selected markers. (A) CD3$^-$CD56$^+$ cells were split into NK cell subsets based on CD56 and CD16 expression (B) CD3$^+$ CD4$^+$ T cells were split into CD4$^+$ T cell subsets based on CD127, CD25, HLA-DR, CD45RA and CCR7 expression; Tconv = conventional T cells, CM = central memory, EM = effector memory, Tregs = regulatory T cells (C) CD3$^+$ CD8$^+$ T cells were split into CD8$^+$ T cell subsets based on CD45RA and CCR7 expression; CM = central memory, EM = effector memory (D) CD3$^-$ CD19$^+$ B cells were split into B cell subsets based on CD27, IgD, IgM, CD24, CD38 expression; Mem = memory (E) CD3$^+$ T cells were split

into subsets based on CD56, γδTCR, CD4 and CD8 expression; DP = double-positive, DN = double negative.

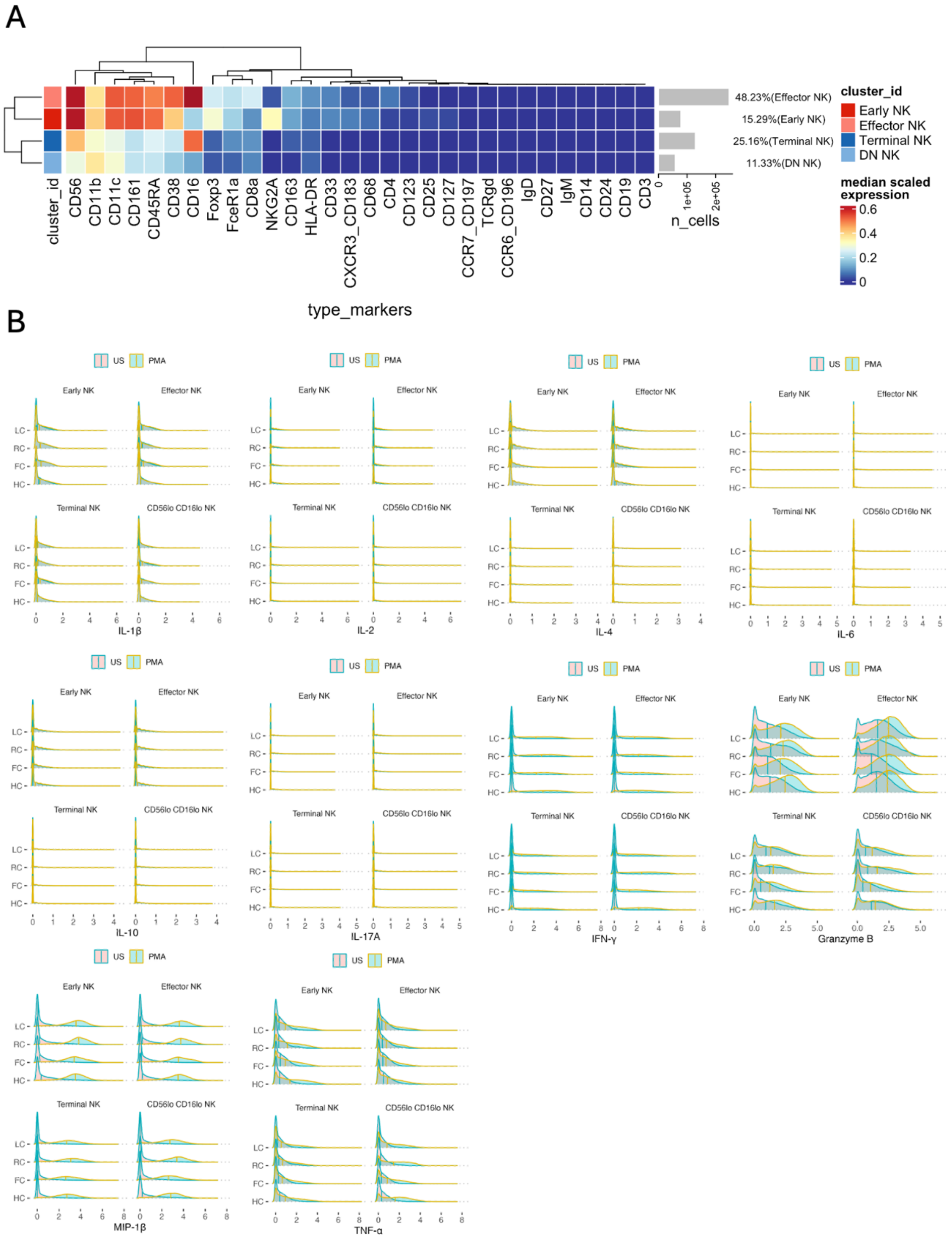

Figure S3. NK cells marker expression. (A) Heatmap showing median scaled expression of surface markers in four NK cell subsets; DN NK = CD56^{lo} CD16^{lo} NK cells (B) NK cell cytokine response. Marker histograms for cytokine expression in four NK cell subsets before and after stimulation; Pink = unstimulated; green = PMA/iono.

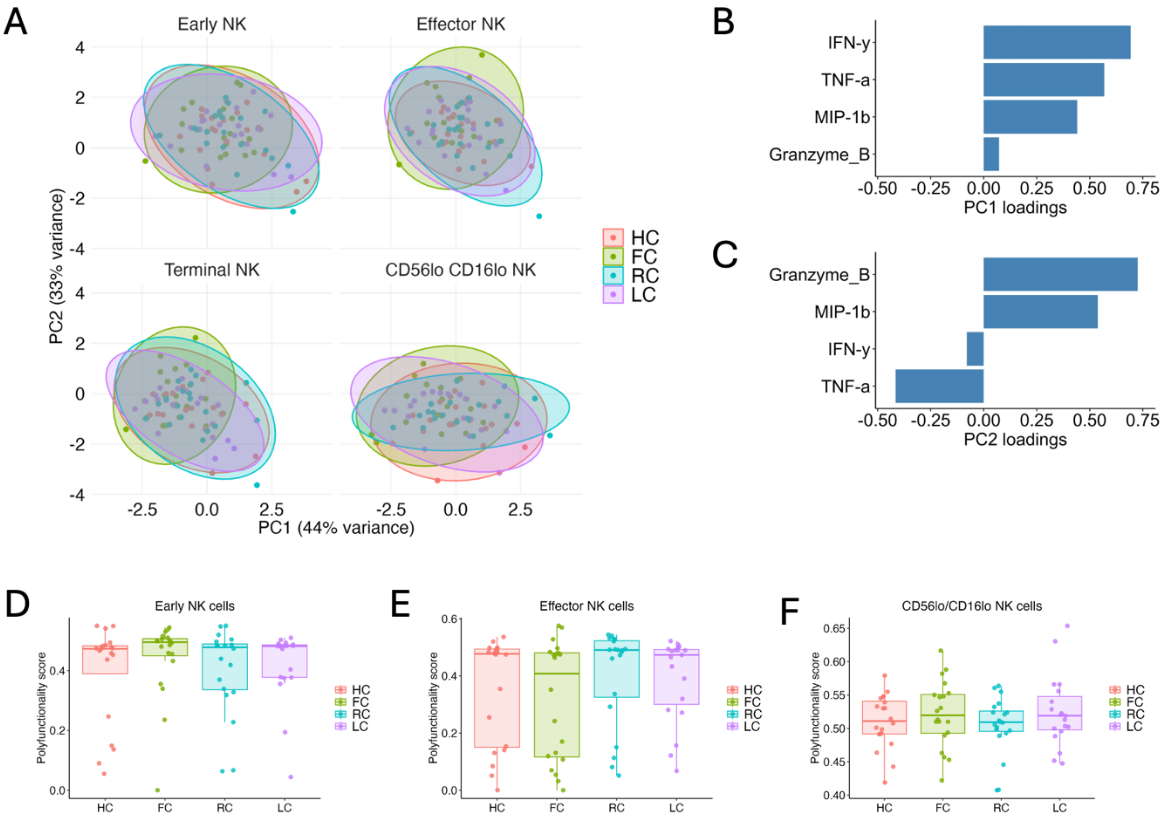

Figure S4: NK cells cytokine response. (A) PCA plot of NK cells after PMA/iono stimulation, per NK cell subset, colored per group (red = HC (n=20), green = FC (n=20), blue =RC (n=20), purple = LC (n=19) with 95% confidence ellipse. (B + C) Loadings of principal components PC1 and PC2 contributing to overall variability in PCA for NK cells overall. (D-F) Polyfunctionality calculated by COMPASS for each sample's response to PMA/iono stimulation, per NK cell subset. Box plots of polyfunctionality score (PFS) in HC (red), FC (green), RC (blue), LC (pink) in response to PMA/iono-stimulation. Pairwise differences were based on Kruskal-Wallis test followed by Dunn's post hoc test.

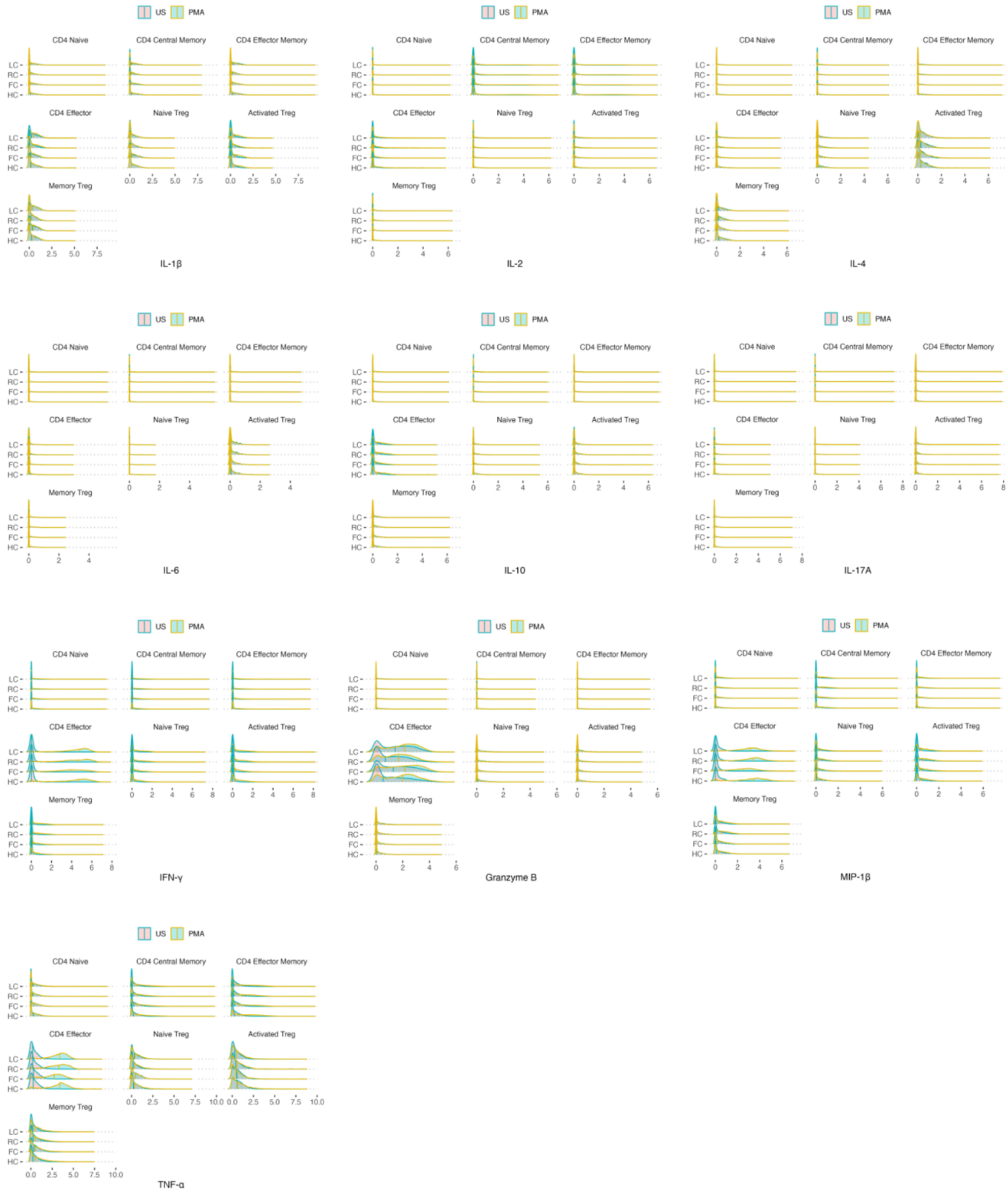

Figure S5: CD4+ T cells cytokine response. Marker histograms for cytokine expression in major CD4+T cell subsets before and after stimulation; Pink = unstimulated; green = PMA/iono.

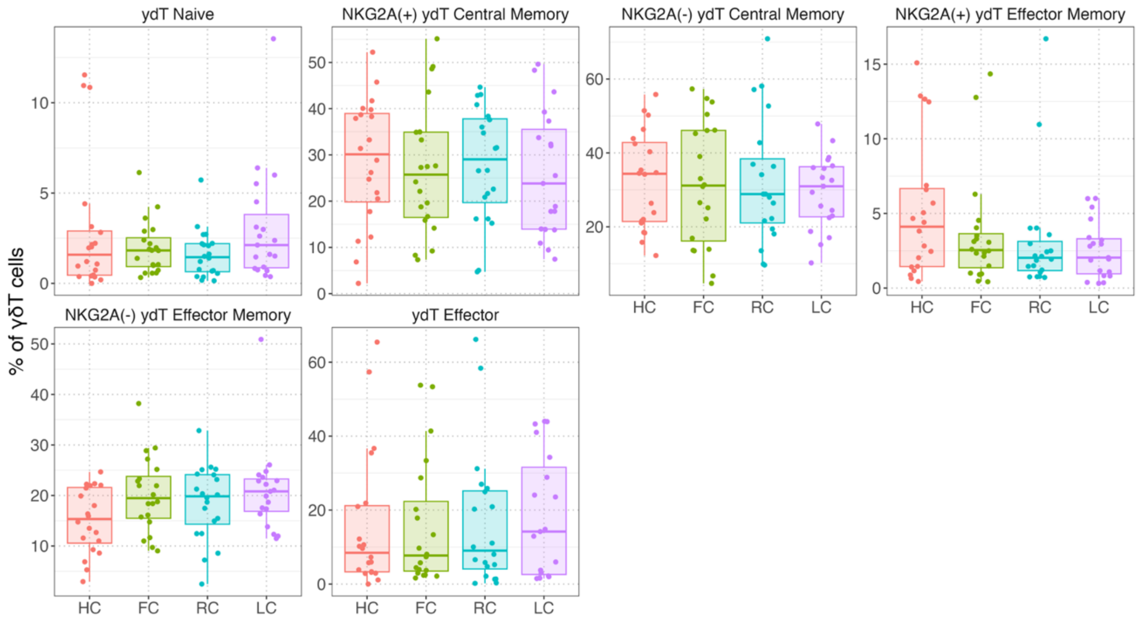

Figure S6: Frequencies of γδT cell subsets. Boxplots with individual levels (dots) display γδT cell subsets frequencies (% of γδT cells) in each group (LC, RC, FC, HC). Negative binomial distribution was used to evaluate differences in mean frequencies in pairwise comparisons between groups. P-values were FDR-adjusted using Benjamini-Hochberg correction. Horizontal bars show statistically significant differences between two groups with *, **, ***, **** = p values of < 0.05, 0.01, 0.001, 0.0001. Box plots: upper, lower and center box lines represent upper quartile, lower quartile and mean.

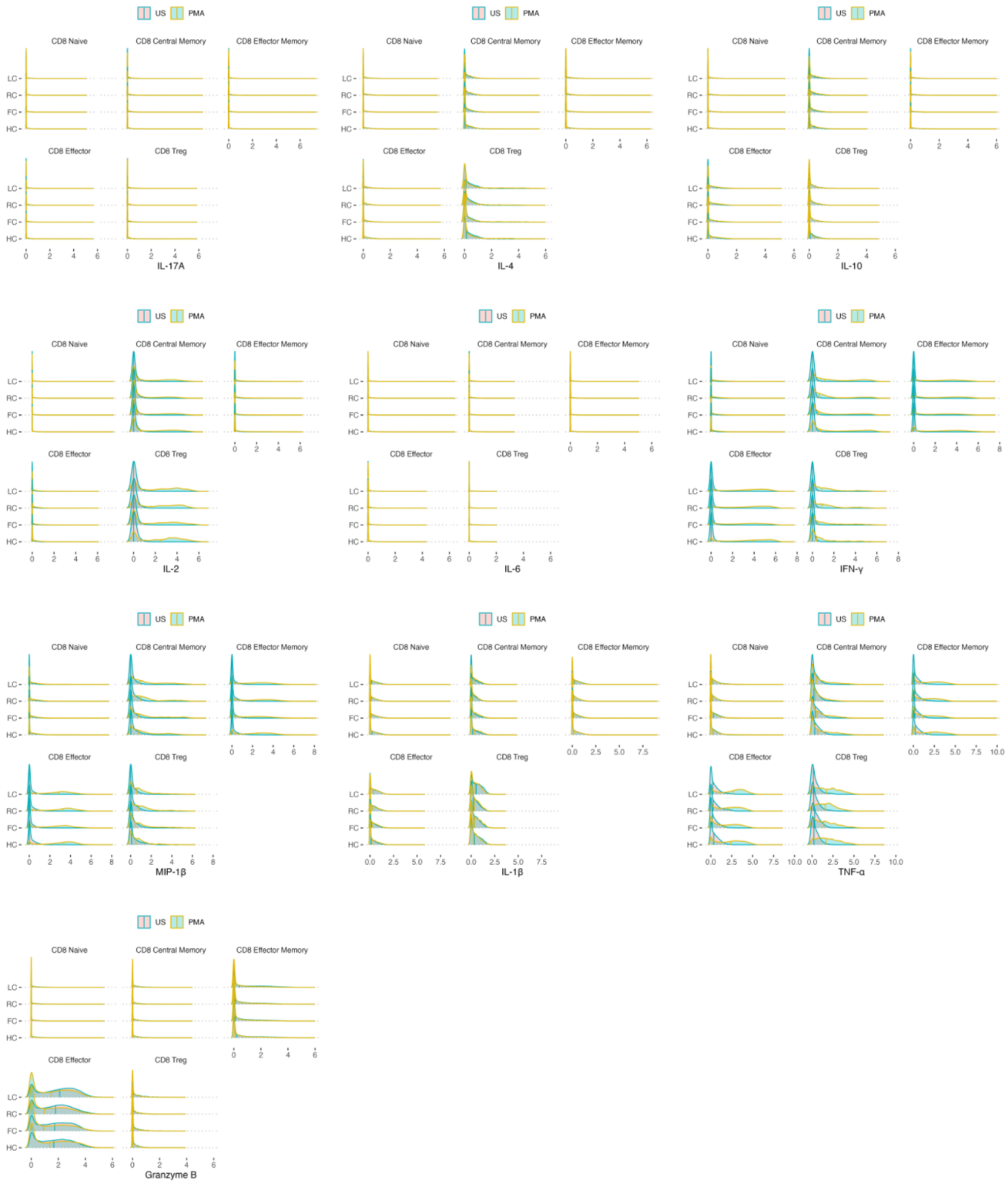

Figure S7: CD8$^+$ T cells cytokine response. Marker histograms for cytokine expression in major CD8$^+$ T cell subsets before and after stimulation; Pink = unstimulated; green = PMA/iono.

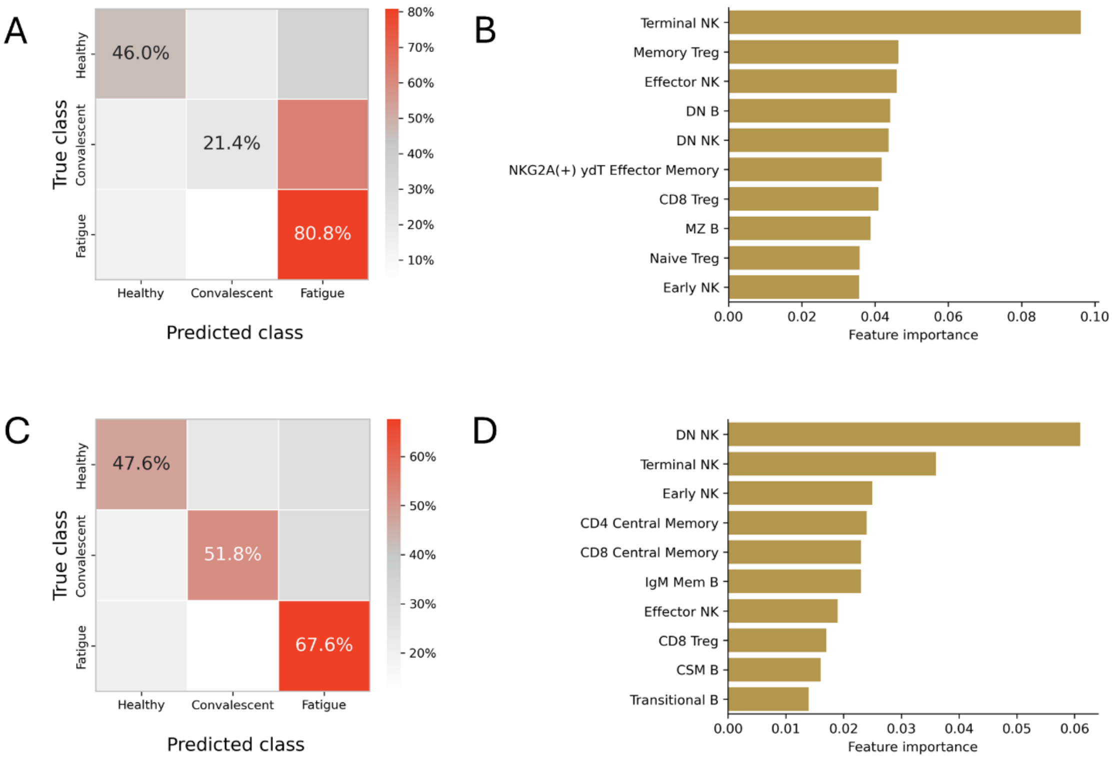

Figure S8: Machine learning-based classification. (A) Confusion matrix showing classification accuracy achieved by Random Forest. Average accuracy scores for 40 iterations are displayed here. (B) Variable importance plot (VIP) showing immune cell subsets and their importance for prediction accuracy obtained by Random Forest. (C) Confusion matrix showing classification accuracy achieved by Linear SVC. Average accuracy scores for 40 iterations are displayed here. (D) Feature coefficients showing immune cell subsets and their importance for prediction accuracy obtained by Random Forest.

Table S1. Adherence to WHO Post COVID-19 condition (PCC) and/or Post-infective fatigue syndrome (PIFS) criteria in the fatigued control (FC) group.

Participant ID	Adherence to WHO PCC criteria (Yes/No)	Adherence to PIFS criteria (Yes/No)	Supplemental information
FC1	Yes	No	Insufficient number of severe symptoms = does not fulfill PIFS case definition
FC3	No	No	Quality of life is too high = does not fulfill WHO/PIFS case definition, also has chronic disease (restless leg syndrome) and adverse life event (broken romantic relationship)
FC16			
FC9	Yes	No	Insufficient number of severe symptoms = does not fulfill PIFS case definition
FC14	No	No	Quality of life is too high = does not fulfill WHO/PIFS case definition
FC5	Yes	No	Insufficient number of severe symptoms = does not fulfill PIFS case definition
FC6	No	No	Sufficient symptoms, but other state: anxiety symptoms, adverse life event (stomach problems) and medication (Betmiga, Vesicare = anticholinergic effect)
FC7	No	No	Sufficient symptoms, but other state: anxiety symptoms and adverse life event (recent new diagnosis: IBS)
FC17	Yes	No	No fatigue at baseline = does not fulfill PIFS case definition
FC19	No	No	Sufficient symptoms, but other state: anxiety symptoms and chronic disease (autism)
FC2	Yes	Yes	
FC18	Yes	Yes	
FC21	Yes	No	Insufficient number of severe symptoms = does not fulfill PIFS case definition
FC10	Yes	No	Insufficient number of severe symptoms = does not fulfill PIFS case definition
FC13	No	No	Sufficient symptoms, but other state: anxiety symptoms and adverse life event (not specified)
FC12	No	No	Quality of life is too high = does not fulfill WHO/PIFS case definition
FC8	Yes	No	Insufficient number of severe symptoms = does not fulfill PIFS case definition
FC11	No	No	Sufficient symptoms, but other state: anxiety/depression symptoms, and adverse life event (broken romantic relationship)
FC20	No	No	Sufficient symptoms, but other state: chronic disease (migraine, psoriasis)
FC4	Yes	Yes	Slightly elevated BNP does not explain symptoms

Ext.Data Table 1. The FC group contains SARS-CoV-2 negative individuals with fatigue symptoms. These were selected based on "no missing data", "female gender", "CFQ caseness" (sum binary CFQ > or equal to 4). These inclusion criteria are the same as for the fatigued COVID19 cases (LC group), with the exception that we could not enforce WHO Post COVID19 and PIFS/Fukuda caseness due to a lack of eligible samples to reach desired sample size. The FC group is therefore composed of two main subcategories: 1) those who comply with WHOPost COVID19 and/or PIFS/Fukuda case criteria and 2) those who do not comply with these criteria. For the first subcategory, the compliance with the criteria implies "exclusion of other states that may explain persistent symptoms". Hence, we did not find any clinical cause for the fatigue in this subcategory when scrutinizing our collected data, as detailed in the publication by Selvakumar et al. However, for the second subcategory, we either found a clinical cause for the fatigue or the persistent symptoms were not severe enough to comply with the case definitions.

Table S2. Mass cytometry panel.

Sl No.	Mass channel	Target	Clone	Catalogue No.	Supplier
1	**89Y**	CD45 Barcode	HI30	3089003B	Standard BioTools
2	**106Cd**	CD45 Barcode	HI30	3106001B	Standard BioTools
3	**110Cd**	CD45 Barcode	HI30	3110001B	Standard BioTools
4	**111Cd**	CD19	HIB19	302247	Biolegend
5	**112Cd**	CD4	RPA-T4	300541	Biolegend
6	**113Cd**	CD8a	RPA-T8	301053	Biolegend
7	**114Cd**	HLA-DR	L243	307651	Biolegend
8	**116Cd**	CD3	UCHT1	300443	Biolegend
9	**127I**	IdU	--	201127	Standard BioTools
10	**141Pr**	CD196 (CCR6)	11A9	3141014A	Standard BioTools
11	**142Nd**	IL-1b	H1b-27	511605	Biolegend
12	**143Nd**	CD123	6H6	3143014B	Standard BioTools
13	**144Nd**	IgD	IA6-2	348235	Biolegend
14	**145Nd**	CD163	GHI/61	3145010B	Standard BioTools
15	**146Nd**	TNFa	Mab11	3146010B	Standard BioTools
16	**147Sm**	CD11c	Bu15	3147008B	Standard BioTools
17	**148Nd**	CD16	3G8	3148004B	Standard BioTools
18	**149Sm**	CD25 (IL-2R)	2A3	3149010B	Standard BioTools
19	**150Nd**	FceR1a	AER-37 (CRA-1)	3150027B	Standard BioTools
20	**151Eu**	CD14	M5E2	3151009B	Standard BioTools
21	**152Sm**	TCRgd	11F2	3152008B	Standard BioTools
22	**153Eu**	IgM	MHM-88	314527	Biolegend
23	**154Sm**	IL-6	MQ2-13A5	3154011B	Standard BioTools
24	**155Gd**	CD27	L128	3155001B	Standard BioTools
25	**156Gd**	CD183 (CXCR3)	G025H7	3156004B	Standard BioTools
26	**158Gd**	IL-2	MQ1-17H12	3158007B	Standard BioTools
27	**159Tb**	CD197 (CCR7)	G043H7	3159003A	Standard BioTools
28	**160Gd**	MIP1beta	D21-1351	3160013B	Standard BioTools
29	**161Dy**	IL-17A	BL168	3161008B	Standard BioTools
30	**162Dy**	Foxp3	PCH101	3162011A	Standard BioTools

31	**163Dy**	IL-4	MP4-25D2	3163011B	Standard BioTools
32	**164Dy**	CD161	HP-3G10	3164009B	Standard BioTools
33	**165Ho**	CD127 (IL-7Ra)	A019D5	3165008B	Standard BioTools
34	**166Er**	IL-10	JES3-9D7	3166008B	Standard BioTools
35	**167Er**	CD24	ML5	311127	Biolegend
36	**168Er**	IFNg	B27	3168005B	Standard BioTools
37	**169Tm**	CD159a (NKG2A)	Z199	3169013B	Standard BioTools
38	**170Er**	CD45RA	HI100	3171010B	Standard BioTools
39	**171Yb**	CD68	Y1/82A	3171011B	Standard BioTools
40	**172Yb**	CD38	HIT2	3172007B	Standard BioTools
41	**173Yb**	Granzyme B	GB11	3173006B	Standard BioTools
42	**174Yb**	CD279 (PD-1)	EH12.2H7	31740020B	Standard BioTools
43	**175Lu**	CD33	HIM3-4	NB500-508	Novus Bio
44	**176Yb**	CD56	NCAM16.2	3176008B	Standard BioTools
45	**191Ir**	DNA	--	201192B	Standard BioTools
46	**193Ir**	DNA	--	201192B	Standard BioTools
47	**194Pt**	L/D Cisplatin	--	201194	Standard BioTools
48	**195Pt**	CD45 Barcode	HI30	3195001B	Standard BioTools
49	**196Pt**	CD45 Barcode	HI30	3196001B	Standard BioTools
50	**198Pt**	CD45 Barcode	HI30	3198001B	Standard BioTools
51	**209Bi**	CD11b	ICRF44	3209003B	Standard BioTools

Table S3. Comparison of frequencies of the major immune cell subsets identified by manual merging of SOM clusters (numbers indicate FDR adjusted p-values from pairwise contrasts of a generalized linear model fit). Highlighted cells indicate p<0.05.

Cell subset	HC vs. FC	HC vs. RC	HC vs. LC	FC vs. RC	FC vs. LC	RC vs. LC
**Early NK**	**0.03771932**	0.52633104	**0.01475625**	0.27265435	0.69605656	0.10206515
**Effector NK**	**0.02760155**	0.52633104	**0.03827756**	0.27265435	0.92693014	0.10206515
**Terminal NK**	**1.74E-05**	**0.00211572**	**3.64E-07**	0.27265435	0.69605656	0.11432091
**CD56loCD16lo NK**	**0.01972032**	0.72433821	0.07877373	**0.0134642**	0.69605656	0.17629084
**Naïve B**	0.77515753	0.7163558	0.65919797	0.93628429	0.96598924	0.93469234
**Transitional B**	0.07292382	0.7163558	0.17220064	0.7690375	0.96598924	0.57741749
**Founder B**	0.77515753	0.62370231	0.55356818	0.93628429	0.96598924	0.93469234
**IgM Mem B**	0.77515753	0.62370231	0.55356818	0.93628429	0.96598924	0.93469234
**IgD Mem B**	0.74868957	0.7163558	0.24300429	0.93628429	0.96598924	0.57741749
**CSM B**	0.77515753	0.7163558	0.65919797	0.93628429	0.96598924	0.93469234
**MZ B**	0.13132302	0.44378298	**0.01490837**	0.93628429	0.96598924	0.57741749
**DN B**	**0.03152302**	0.44378298	**0.01513647**	0.7690375	0.96598924	0.57741749
**CD4 Naïve**	0.47802001	0.073619312	0.90100684	0.35024266	0.72537391	0.08136444
**CD4 Central Memory**	0.74415203	0.073619312	0.90100684	0.140522	0.72537391	**0.03597521**
**CD4 Effector Memory**	0.47802001	0.403901077	0.90100684	0.79381727	0.78282077	0.82903023
**CD4 Effector**	0.47802001	0.245940122	0.90100684	**0.0457132**	0.78282077	0.08136444
**Naïve Treg**	0.74415203	0.135325923	0.90100684	0.21559669	0.72537391	**0.03597521**
**Memory Treg**	0.25784347	0.245940122	0.90100684	**0.00416625**	0.72537391	0.10469367
**Activated Treg**	0.74415203	0.314812445	0.96275699	0.50890491	0.78282077	0.34551175
**CD8 Naïve**	0.87149927	0.70717428	0.87594581	0.9220786	0.9584709	0.72161317
**CD8 Central Memory**	0.8414734	0.64281345	0.84942155	0.16201649	0.9584709	0.07551011
**CD8 Effector Memory**	0.8414734	0.71162058	0.87594581	0.9220786	0.9584709	0.72161317
**CD8 Effector**	0.93866457	0.76764738	0.84942155	0.9220786	0.9584709	0.72161317
**CD8 Treg**	0.8414734	0.1205256	0.84942155	**0.00928207**	0.9584709	**0.0129223**
**ydT Naive**	0.38989891	0.24360464	0.86061711	0.90082433	0.61975071	0.18241634
**NKG2A(+) ydT Central Memory**	0.99858421	0.98129314	0.82625824	0.90082433	0.80000094	0.78256016
**NKG2A(-) ydT Central Memory**	0.99858421	0.98129314	0.82625824	0.90082433	0.80000094	0.78256016
**NKG2A(+) ydT Effector Memory**	0.38989891	0.24360464	0.07011538	0.90082433	0.62626004	0.78256016
**NKG2A(-) ydT Effector Memory**	0.27356582	0.30577636	0.07011538	0.90082433	0.80000094	0.78256016
**ydT Effector**	0.99858421	0.98129314	0.82625824	0.90082433	0.80000094	0.78256016
